# Study protocol for a pilot study for Remote ADHD Monitoring Program (RAMP) for children in rural areas

**DOI:** 10.1371/journal.pone.0337802

**Published:** 2025-12-02

**Authors:** Claire A. MacGeorge, Matthew Henry, Hannah A. Ford, Lacy Malloch, Emily Fratesi, Shannon Cabaniss, Jaime Baldner, Melody Greer, Kristin Gaffney, Milan Bimali, Preetha Abraham, Linda Y. Fu, P. Songthip Ounpraseuth, Christine B. Turley

**Affiliations:** 1 Medical University of South Carolina, Charleston, South Carolina, United States of America; 2 University of Arkansas for Medical Sciences, Little Rock, Arkansas, United States of America; 3 University of Mississippi Medical Center, Jackson, Mississippi, United States of America; 4 National Institutes for Health, Bethesda, Maryland, United States of America; 5 Wake Forest University School of Medicine, Atrium Health Levine Children’s, Charlotte, North Carolina, United States of America; PLOS: Public Library of Science, UNITED KINGDOM OF GREAT BRITAIN AND NORTHERN IRELAND

## Abstract

**Background:**

Attention-deficit/hyperactivity Disorder (ADHD) is the most common neurobehavioral condition of childhood and can be controlled with stimulant medication. Evidence-based guidelines endorse use of standardized ADHD symptom reports to facilitate medication titration to therapeutic dosage. Children living in under-resourced areas experience barriers to receiving this recommended evidence-based care. The Remote ADHD Monitoring Program (RAMP) uses a text-based platform to relay symptom reports from caregivers and teachers to healthcare providers. This pilot study is a feasibility study examining intervention uptake. It compares the submission of structured symptom reports in those children enrolled in RAMP compared to usual care as well as utilization of the RAMP platform by providers.

**Methods:**

This paper describes the protocol to evaluate the feasibility of deploying RAMP in practices serving rural or underserved children. We will recruit 36 dyads from 4 practices in 2 separate states. Each dyad will include a caregiver and their child aged 5–11 years with a diagnosis of ADHD who is starting or reinitiating stimulants. Dyads will be randomized 1:1 to receive the RAMP intervention or usual care with attention controls. Our primary outcome is number of symptom reports (paper assessments in control arm and RAMP reports in intervention arm) per participant that are completed by caregivers and teachers and returned to providers. Our secondary outcome is proportion of submitted RAMP reports that are reviewed by providers.

**Discussion:**

As telehealth use increases, it is critical that we improve access to high quality care for children with chronic conditions. Leveraging technology may be a meaningful approach to improve efficiency in optimizing medication management. This pilot study tests a text-based platform designed to improve communication between the caregivers and teachers of children with ADHD and health care providers. If successful, a future trial will examine the effectiveness of the RAMP intervention on improvement in symptoms.

**Trial registration:**

ClinicalTrials.gov NCT06743425.

## Introduction

The 2019 American Academy of Pediatrics (AAP) Clinical Practice Guidelines state that stimulant medications for attention-deficit/hyperactivity disorder (ADHD) can be effectively titrated on a 7-day basis [1]. Studies, including the Multimodal Treatment Study of Children with ADHD, have shown that rapid stimulant medication titration combined with close communication between providers and teachers yields better outcomes than behavioral intervention alone or standard community care [2].

Despite these established guidelines, children in rural settings are less likely to receive timely follow-up and be prescribed stimulant medications than their urban counterparts [3,4]. These differences are driven by a combination of patient- and provider-level barriers. At the patient level, rural families often have limited access to pediatric behavioral health services. They face greater burdens related to distance from care and management of chronic health conditions and overall have poorer health status [5]. These realities make it difficult for caregivers to consistently engage in the exchange of symptom information with providers and refine treatment plans to align with ADHD best practice recommendations. On the provider side, clinicians in rural areas also face challenges such as limited information technology infrastructure, varied Electronic Medical Record (EMR) systems, and a lack of pediatric behavioral health specialists, all of which hinder their ability to follow ADHD guidelines effectively [6].

ADHD diagnosis and treatment require multiple coordinated steps, including identifying behavioral or academic problems, collecting standardized ADHD symptom reports (such as the Vanderbilt ADHD Rating Scales (VARS), from both parents and teachers), prescribing and titrating medication, and conducting regular follow-up [7,8]. These steps require a high degree of caregiver engagement, and disruptions in this process —especially common in rural areas—can significantly delay symptom control and worsen long-term outcomes.

Structured communication tools, such as electronic portals, have improved ADHD care in urban settings by streamlining communication among caregivers, teachers, and providers [9,10]. However, these tools are often based on a single EMR system or embedded in complex technology infrastructures that are less common in rural practices. The traditional paper-based workflow for collecting standardized ADHD symptom reports, exchanged among home, school, and clinic settings via physical handoffs is inefficient, resulting in underuse of these reports for treatment decisions. Research shows that only 50% of community healthcare providers use structured assessments to diagnose children with ADHD, and just 10% use them at follow-up appointments. This causes a significant gap in quality care [11].

To address these issues, this pilot examines the feasibility of the Remote ADHD Monitoring Program (RAMP) intervention. RAMP is a program utilizing a non-EMR-based, text-message-driven platform designed to support structured ADHD symptom communication in the form of RAMP reports in rural settings. The RAMP report includes the publicly available VARS, which is a commonly used, guideline-recommended tool for both diagnosis and management of ADHD for use by parents and teachers (see Appendix 1 in S3 file) [12]. The VARS assessment was initially validated in a referral population with teacher reporters in 1998 and with parent reporters in 2003, and subsequently in a community population with both types of reporters in 2013 [7,8,12,13].

RAMP offers a potential solution to overcoming common obstacles. By using a text-based platform independent of EMR constraints, it offers flexibility across varied rural practice environments. RAMP fits with caregiver preferences for mobile-based interaction and reduces the burden on providers to manually gather assessment data. It also avoids the need for technology investment at the practice level and EMR compatibility issues that often hinder rural practices from adopting new digital tools.

This pilot study aims to evaluate the feasibility of implementing RAMP in practices serving rural or underserved children. The research questions for the pilot project are as follows. First, will caregivers and teachers of patients living in these settings return completed VARS more frequently on the RAMP platform versus on paper? Second, will healthcare providers in practices serving rural or underserved children access the RAMP platform to view VARS returned from caregivers and teachers?

## Materials and methods

### Study design and setting

The pilot is designed as a multi-centered randomized controlled exploratory clinical trial to test the feasibility of implementation of the RAMP intervention. We will seek a total of 4 practices with high percentages of rural or underserved pediatric patients across two states. Rural practices will be defined as those practices with all of the following: caring for >700 children in the study inclusion age group (5–11 years), located in small cities (<50,000 people), and located further than 30 minutes driving distance from a pediatric academic medical center. We included these practices as they experience shortages of pediatric sub-specialists (e.g., developmental-behavioral pediatricians) and mental health providers due to geographic barriers. A driving distance of 30 minutes was used as it is a standard that has been proposed by health departments and used in prior studies [14–16]. Practices caring for underserved children will also qualify to participate if at least 40% of their pediatric patient visits are covered by Medicaid insurance. Practices must use an EMR for generalizability and relevance to future studies; however, RAMP itself is EMR independent.

### Participants

**Inclusion criteria:**

To be eligible to participate in the trial, caregiver/child dyads must attend one of the four participating primary care practices and meet the following inclusion criteria:

The child must

- be under the medical care of a participating provider
- be aged 5–11 years at enrollment
- have a diagnosis of ADHD
- be initiating stimulant medication for treatment for the first time or not have received stimulant medication in the last 3 months
- be attending in-person elementary school

The caregiver must

- be willing and legally able to give consent
- have access to a smartphone
- be English-speaking
- reside with the child at least 3 days per week
- complete an initial symptom assessment prior to starting stimulant treatment and be willing to provide a copy to the study team

**Exclusion criteria:**

- Child must NOT
  - have any severe mental health comorbidity including schizophrenia, bipolar disorder, conduct disorder or hospitalization for any mental health condition.
  - have a severe or uncontrolled neurodevelopmental disorder.
  - be currently receiving, or have previously received, atypical antipsychotic medication treatment
  - be pregnant

### Recruitment, consent and enrollment

**Fig 1:**
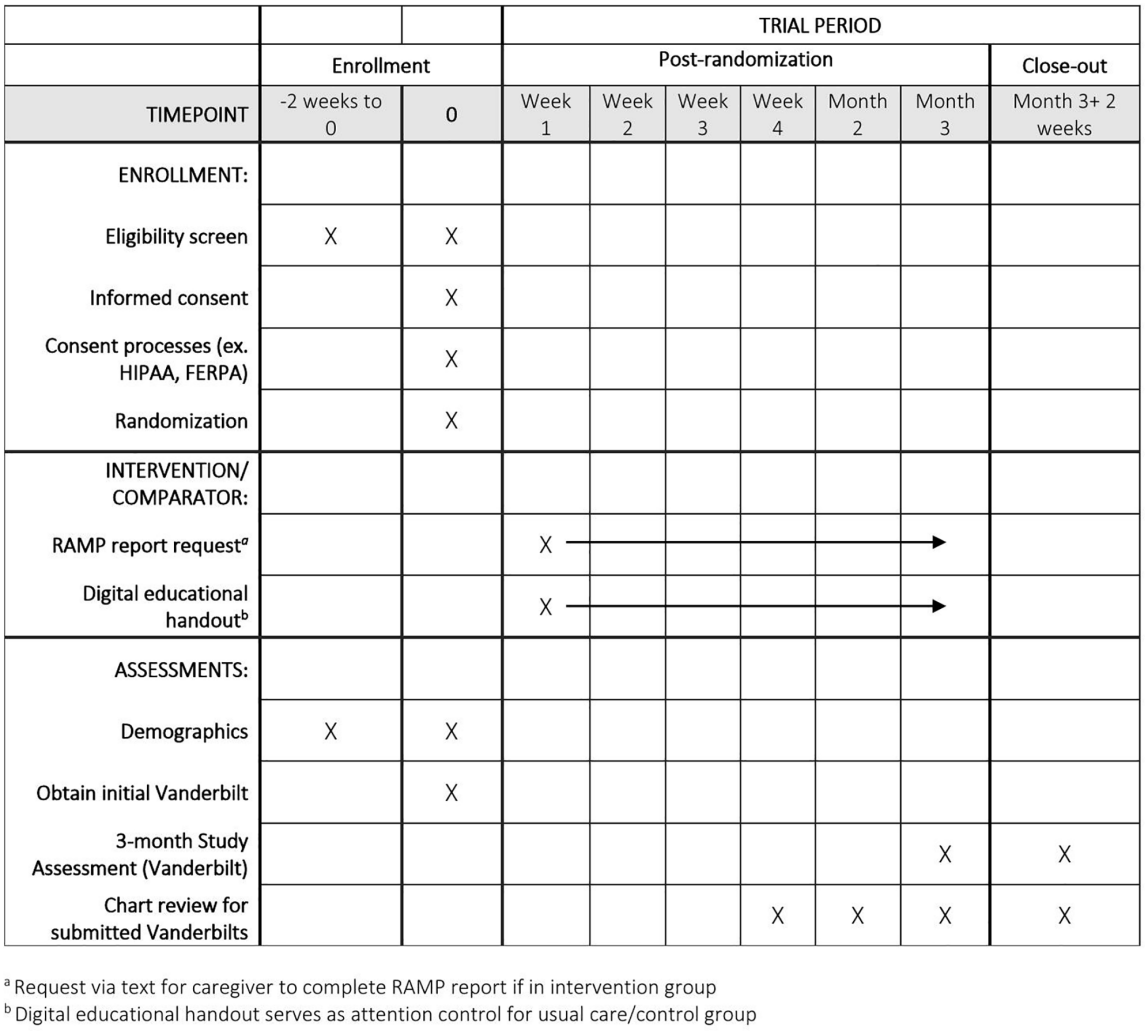
Schedule of Study Activities.

The schedule of activities can be found in Fig 1. In the first phase of the study, the providers at participating practices will be recruited and consented. Research coordinators will work with practice providers to schedule a time for a study presentation and obtain informed consent from providers who care for children with ADHD at the practices that showed expressed interest. Providers will have an opportunity to ask questions and sign the consent documents at that time or to schedule a private consent encounter with a research coordinator.

In the second phase of the study, caregiver and child dyads will be recruited, consented and enrolled. Providers will notify research coordinators of potential participants following an initial evaluation visit or medication reinitiation visit for ADHD. Research coordinators may also search for potential participants in the EMR and contact the provider about the possible participant. A partial Health Insurance Portability and Accountability Act (HIPAA) waiver will be obtained for pre-screening procedures used to identify potential participants. Upon the child’s return visit if the child receives an ADHD diagnosis and is started on stimulant therapy, caregiver/child dyads will receive a study recruitment flyer from the provider and, if amenable, be referred to a research coordinator for review of the study to assess their caregiver/child interest in participating.

Research staff will contact interested caregivers by phone, email, and/or text. If caregivers are unreachable by any of one these methods, research staff will follow up with at least two additional outreach attempts by one or more additional methods within the first five weeks after the child has been first prescribed stimulant medication. After a discussion of the study, if a caregiver does not want to participate, the research coordinator will document the reason for study participation refusal.

For all interested caregivers, who must be legally authorized representatives (LARs) of the child, e.g., the parent or guardian, the study team member will complete the eligibility screening. Enrollment procedures will include orientation to study procedures followed by obtaining written informed consent, HIPAA and Family Educational Rights and Privacy Act (FERPA) authorizations, demographics and baseline medical history. Assent for the child will be waived. The LAR’s consent, for themselves and their child, will be documented per policies of the central IRB. FERPA authorization allows teachers to send ADHD symptom information to the providers in the RAMP platform. Research coordinators will also ask for the caregiver’s permission to obtain the initial diagnostic assessment from the EMR. Eligible caregiver/child dyads will be randomized to either intervention or control group (1:1). Randomization will be performed using a master randomization list stratified by site; block randomization will be used. The master randomization list will be created by the DCOC Biostatistics team using statistical software. Research coordinators will be blind to the allocation until they have enrolled the participant at which time they will access the allocation in the electronic data capture module. No blinding will occur after randomization process.

The study principal investigator will meet with study staff regularly to review study enrollment and retention as well as any relevant protocol changes. A member of the practice staff (nurse, medical assistant or practice manager) will be identified as site champion. Calls with the site champions and ideally at least one healthcare provider at each practice may occur during the enrollment period to keep practice staff informed about enrollment progress. Assistance to a specific practice will be provided if a practice has not been able to enroll at least 4 caregiver/child dyads within 6 weeks of initiating recruitment/enrollment.

At the time of submission for publication, we have not completed recruitment nor generated results. We anticipate that recruitment will occur from March 1, 2025 through November 30, 2025. We will be gathering data on these participants through March 2026 and will generate results in June – July 2026.

### Study procedures

All healthcare providers from participating practices will receive a standard 1-hour didactic session on ADHD best practices and training in the RAMP platform use. Healthcare providers will be asked to provide informed consent because we will be collecting data on their clinical workflow and use of RAMP. Since patients are sometimes treated by multiple members of a practice team, all healthcare providers of a participating practice must agree to enroll in the study for the practice to participate. The site champion will facilitate communication between the site study team and the practice staff.

After provider enrollment and training have concluded, healthcare providers will refer caregivers of patients with a diagnosis of ADHD who are being started on stimulant medication or are re-initiating after being off for 3 months to study staff. Research coordinators will discuss the study, perform eligibility screening and, if eligible, obtain informed consent from the caregiver for participation of caregiver/child dyads. Caregiver/child dyads will be randomized 1:1 to intervention (RAMP) or control (usual care with a digital handout serving as an attention control) groups. Families will be approached until 36 caregiver/child dyads are enrolled (only one child in a family may be enrolled) or the time limit for enrollment is reached.

Each week, progress to target will be evaluated to determine if any practice needs additional resources. As this is a pilot study, some adaptive trial design methods will be employed. Specifically, if after 16 weeks of recruitment, multiple practices do not meet enrollment goals of 2 participants per month, the study team may consider relaxing exclusion/widening inclusion criteria (e.g., include patients undergoing re-titration, and/or widen age criteria).

Enrollment will be paused for 3 months from the last month of school to the start of the new school year (beginning of May to end of July). This will decrease the burden on practices and study staff during the time of the year with the fewest number of ADHD diagnoses. Once enrolled, caregiver/child participants will be randomized to either the intervention or control group and followed for 3 months (plus 2 weeks if needed for return of final VARS). Each group will receive all other concomitant care (additional testing, medication management, in-person follow-up visit scheduling) at the discretion of the health care provider.

For all child participants, research coordinators will periodically review the EMR to record the number of paper VARS returned to a practice during the intervention period. They will also access the RAMP platform to review RAMP reports returned by caregivers/teachers, and complete Provider Review Surveys documenting their review. All caregiver participants will complete a 90-day study VARS as a retention measure. This phase will continue until all enrolled participants have either completed their 3-month study period or have left the study.

### Intervention

The RAMP platform will be a clinical site-specific Research Electronic Data Capture (REDCap) program, a HIPAA-compliant data collection and management tool for clinical trial data [17]. The intervention will include caregiver and teacher prompts from the RAMP platform to complete RAMP reports of inattention and hyperactivity symptoms and a provider dashboard listing returned RAMP reports for enrolled children. RAMP is adapted from a successful text-based behavioral health screening program called Listening to Women used for pregnant women in South Carolina. The program showed improved outcomes compared to traditional care [18]. Listening to Women has been shown to be scalable, cost-effective, and aligned with user preferences for mobile-based communication; these factors make it particularly well-suited for rural settings with limited technological infrastructure [18].

Caregivers and teachers of participants randomized to the intervention group will be asked to complete weekly RAMP reports of the child’s ADHD symptoms for 4 weeks followed by monthly RAMP reports for 2 months. They will receive automatic text message prompts to complete the symptom assessments. Potential concerns about participant adherence to the intervention include the length of the RAMP report and frequency of report completion requests. A text message-based platform with a direct link to radio-button style responses will be used to decrease this burden. Log-in is not required, and the RAMP platform is formatted to be compatible with smart phone-based usage for completion. Caregiver participants will receive automated reminder prompts at 24 and 48 hours if the RAMP report has not been completed. Each RAMP report will be submittable by caregivers for 72 hours after the initial completion request has been texted. Study coordinators will check-in with caregiver participants to inquire about any technical difficulties with the RAMP platform or barriers to completing the forms at approximately 1, 4, and 28 weeks from the time of consent.

Specifically, the RAMP Caregiver report includes all items in the VARS Parent Informant questionnaire, as well as questions about engagement in behavioral health care visits, and progress in educational settings. These domains of information have been selected as they are typically considered by providers making decisions regarding ADHD medication dose adjustment.

Caregivers in the intervention group will also be provided with digital and paper handouts that they will be asked to share with their child’s teachers. Teachers will not be considered participants in this study, hence there will be no communication between study staff and teachers. Teachers will be limited to actions they already perform as standard care (namely submission of ADHD symptom information to health care providers). The handouts include the caregiver-signed FERPA and a secure, unique link to access RAMP teacher reports for a specific child participant. This link will be in the form of a QR code which can be printed on paper or emailed directly to teachers from caregivers. Teachers will be requested to complete the RAMP Teacher report directly into the RAMP platform at the same intervals as caregivers. We will use an approach similar to that used by Power *et al* to collect standard of care information from teachers using the RAMP platform [19]. Similar to RAMP Caregiver reports, completed RAMP Teacher reports will be available for the child’s healthcare provider to view on the RAMP platform. If a child’s teacher does not want to complete RAMP reports, the child will not be removed from the study. If a child has multiple teachers, the caregiver will be asked to request that only one teacher, specifically the one who spends the most time with the child, complete all RAMP Teacher reports.

**Fig 2:**
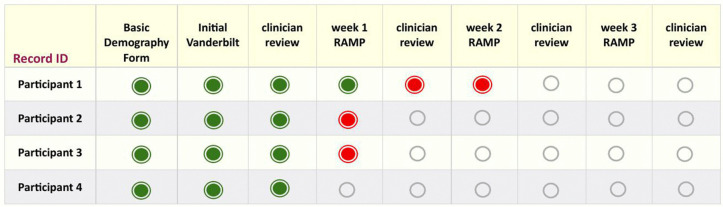
RAMP Dashboard Prototype.

Participating healthcare providers will be alerted by their preferred means (text or email) whenever new caregiver or teacher reports are available for review. Providers will be able to review RAMP reports from a dashboard similar to Fig 2. A red dot will indicate the availability of an unreviewed RAMP report. Opening a report triggers the RAMP platform to ask the provider to complete the Provider Survey. The Provider Survey consists of two items, one to confirm they have reviewed the report and two to document the date of review. While providers will have access to the RAMP platform at all times, they will only be asked to complete one Provider Survey per week for any given week that there is a RAMP report available to review. Research coordinators will be able to monitor report completion by caregivers and monito report reviews by providers.

### Control

Digital education handouts will be sent to all caregivers in the control group on a weekly basis for 4 weeks, and then monthly for 2 months—i.e., on the same schedule as RAMP report requests are sent to caregivers in the intervention group. Digital education handouts will be sent through email and will cover a range of general pediatric topics such as nutrition, exercise, and screen time.

### Engagement and retention strategies

Both provider and caregiver participants will be compensated for their time and effort for the study through ClinCards (or site’s preferred method). Providers will receive $100 after completing the provider orientation and $100 at the study conclusion. Caregivers will be compensated for their participation in the study after completing the enrollment visit ($100), and after completing the 90-day study VARS assessment ($100). Maximum provider and caregiver compensation is $200. Practices will receive a $4000 stipend for participation.

All caregiver participants will complete a 90-day study VARS assessment as a retention measure. If the 90-day study assessment is not received within 10 days of the initial completion request, the research coordinator will follow up with a phone call and offer to provide the assessment again using the participant’s preferred method. This follow-up may be repeated one additional time after an additional 10 days.

### Statistical considerations/Analysis

The statistical team will conduct all statistical analyses following the statistical principles for clinical trials as specified in ICH Statistical Principles for Clinical Trials (ICH Topic E9). The study team will present study data and summary tables for the overall study and by study site.

Descriptive data summaries for continuous data will include mean and standard deviation or median and interquartile range, as appropriate. The team will also summarize categorical data by using frequency and proportion. Data will be inspected to assess eligibility, out-of-range values, skewness, and missingness. Analysis methods appropriate to the data characteristics will be applied.

### Primary endpoint

Determining the utilization of clinical ADHD assessments (paper assessments in control group and RAMP reports in intervention group) in practices serving rural or underserved children will be a key parameter necessary for conducting a future multi-site efficacy trial. Utilization will be determined by assessing the numbers of completed caregiver and teacher reports in each group. Reports included in the primary outcome analysis will be any clinical assessments – such as paper VARS or RAMP reports – that are submitted to a provider. (The 90-day study assessment will be collected only to ensure feasibility of their collection in a full-scale trial.) Any paper VARS assessments submitted by intervention group participants will be counted separately from the RAMP reports for sensitivity analysis. Data analysis will report mean, standard deviation, median, and interquartile range for each utilization measure overall. Additionally, we will develop a Poisson regression model to compare the number of returned structured reports across the groups. We will report the expected mean number of reports in each group along with their difference with 95% confidence interval (CI).

### Secondary endpoint

The secondary endpoint, proportion of Provider Review Surveys with at least one available RAMP report where review is documented, is only relevant to the subset of participants randomized to the RAMP intervention group. There is no formal hypothesis testing for this secondary objective. The proportion of Provider Review Surveys with at least one available RAMP Caregiver or Teacher report where review is documented will be estimated along with the corresponding 95% confidence interval using the Wilson score interval or other method appropriate to the data characteristics.

### Sample size

Up to 36 caregiver/child dyads and 20 providers will be enrolled. Our proposed sample size is consistent with the recommendations of Whitehead and colleagues for pilot studies [20]. Their derived new stepped rules of thumb for a pilot trial sample size vary depending on an estimated standardized effect size for the full-scale, efficacy trial; therefore, if we assume a medium Cohen’s effect (i.e., 0.3 ≤ δ ≤ 0.7) and 90% power for the full-scale trial, a sample size of 36 caregiver/child dyad participants with 18 randomized to each of the two arms in the pilot study is sufficient.

With a focus on participant recruitment and adherence, a sample size of 36 caregiver/child dyads allows the study team to estimate an expected recruitment rate of 25% (±12%) of those approached and adherence rate of 50% (±14%) for those enrolled with a confidence level of 90% [21]. We expect the rate of return of the RAMP reports to be a comparable rate to the general survey response rate of 52.7%, though it may be higher as the RAMP platform aims to function as an engagement tool [22].

### Data management and monitoring

Data management will be overseen by the ISPCTN Data Coordinating Center (DCOC) at the University of Arkansas for Medical Sciences. The clinical database management system (CDMS) will be built using a validated electronic data capture system, with an audit trail, that is fully compliant with 21 CRF Part 11. The CDMS will be designed, developed, validated and managed by the DCOC. Edit checks will be developed by the DCOC to check data values for inconsistencies, including missing values, values out of range, invalid and illogical data. The electronic data capture (EDC) system will have a discrepancy management system to track the status of the electronic edit checks and allows for manually created queries. Status, Exception and Safety reports will be generated and distributed to the Protocol Team weekly to monitor the progress and outstanding data for the study.

Study progress and safety will be reviewed monthly (and more frequently if needed) by the study investigators for patient recruitment and retention rates and for unsolicited reports of adverse events. Because this study is of an educational intervention, study-related adverse events (AEs) and serious adverse events (SAEs) are not expected. Given the minimal risk of the study, the study team will not solicit AEs or SAEs. However, the study team will provide all participants with a phone number to report AEs and SAEs.

Safety oversight will be under the direction of a Data and Safety Monitoring Board (DSMB) composed of individuals with the appropriate expertise, including pediatrics, clinical trial management and biostatistics. Members of the DSMB will be independent from the study conduct and free of conflicts of interest, or measures should be in place to minimize perceived conflicts of interest. The initial DSMB meeting will occur before the start of the trial to discuss the protocol and the DSMB Charter, which includes data review in open and closed table shells, the Data and Safety Monitoring Plan, definition of a quorum, and guidelines for monitoring the study. The DSMB will operate under the rules of an approved charter that will be written and reviewed at the organizational meeting of the DSMB, and each data element that the DSMB needs to assess will be clearly defined. The DSMB will provide its input to the NIH. Following the initial meeting, the DSMB will meet at least twice a year thereafter, per the DSMB charter, to assess safety and study enrollment. Adverse events will be tabulated by type, severity and relatedness to study treatment at the event level and participant level for review by the DSMB at the site of DSMB meetings.

### Ethics and dissemination

This study, protocol, and all instruments, including the informed consent document, have been approved by the Institutional Review Board (IRB) of the University of Arkansas Medical Sciences (UAMS) (FWA00001119). The UAMS IRB is part of the UAMS Human Research Protection Program and is fully accredited by the Association for the Accreditation of Human Research Protection Programs (AAHRPP). The outcomes of this study will be published and presented at national and/or international conferences as well as patient-facing informational websites. All data presented will be de-identified. The resources developed in this study will be made publicly available.

## Discussion

As telehealth increasingly becomes more common, we have the opportunity to improve access to high quality care for children with ADHD in rural and underserved areas by leveraging technology. This pilot study tests a text-based platform designed to improve communication between the caregivers and teachers of children with ADHD and the children’s health care providers. By focusing on feasibility and process outcomes, this pilot study will inform whether the RAMP intervention can be embedded into routine primary care practice in a larger efficacy trial. If successful, findings from this pilot will inform approaches to enhance provider and caregiver adherence to the intervention. A future trial would focus on improvement in symptoms (efficacy).

## Supporting information

S1 File RAMP SPIRIT Checklist. (DOCX)

S2 File RAMP Protocol Version 4, approved June 25, 2025. (PDF)

S3 File Appendix 1. (PDF)
