# Supplementary material for: Study protocol for a pilot study for Remote ADHD Monitoring Program (RAMP) for children in rural areas
Source: PLoS One. 2025 Dec 2;20(12):e0337802. doi: 10.1371/journal.pone.0337802 (PMC12671750; doi:10.1371/journal.pone.0337802)
Supplement: S2 File — (PDF) [file pone.0337802.s002.pdf]

A Pilot Study of a Remote Attention-Deficit/Hyperactivity Disorder Monitoring Program (RAMP) for Children in Rural Areas

UAMS IRB Protocol Number: 276280

Version Number: V-04

Date: 25-June-2025

National Clinical Trial (NCT) Identified Number: NCT06743425

DCOC Principal Investigator: Song Ounpraseuth, PhD

(ISPCTN DCOC at UAMS)

Principal Investigator: Claire MacGeorge, MD, MSCR

Medical University of South Carolina

Sponsor: ISPCTN DCOC

Funded by: NIH

Summary of Key Changes from Version-03 to Version-04:

Affected Section(s)	Summary of Revisions Made from V-03 to V-04	Rationale
1.1, 4.2, 5.1	Eligibility criterion that the child has not received stimulant medication for the last 6 months reduced to the last 3 months.	Increase the number of eligible children.
1.3, 5.5.1	"Caregiver/child participant dyads will be enrolled within 2 weeks of the child being prescribed stimulants," was changed to, "Caregiver/child participant dyads will be enrolled within 5 weeks (35 days) of the child being prescribed stimulants."	Increase the number of eligible children.
1.1, 1.3, 4.1,	Study duration increased from 10 months to 17 months, with a 3 month enrollment pause from May 6, 2025 through July 31, 2025, while children are out of school.	New ADHD diagnoses are typically infrequent during the summer.
9.3, Population for analysis	"To evaluate symptom improvement of the participants in the RAMP intervention, the study will have two analysis populations," was changed to, "To evaluate completion of clinical assessments and provider acceptability, the study will have two analysis populations:"	Correction

Summary of Key Changes from Version-02 to Version-03:

Affected Section(s)	Summary of Revisions Made from V-02 to V-03	Rationale
1.1, Synopsis	Added that child participants who have not received stimulants for the last 6 months may be eligible.	Added detail. Change made in V-02.
1.2.1 Schema	Text in lower right corner of schema changed from "Mid-point and Final Study Assessments," to, "3 month Study Assessment."	Correction. Change made in V-02.
5.1, 5.5.4	"Participating practices will receive an incentive of $2000 for their time and engagement in the study," changed to, "Participating practices will	Practices will be compensated for their time.

	receive compensation of $4000 for their time and engagement working on the study."	
8.3.1, Definition of Adverse Events	Added, "This study will only report AEs for the child participant due to the negligible risk to providers and caregiver participants."	Engaging in online activities is commonplace today.
8.3.4, Time Period and Frequency for Event Assessment and Follow-up	"For their child," added to the following sentence: Clinic providers participating in this study will be asked to remind caregiver participants to report any medication side effects or events that caused them to seek medical assistance or hospitalization for their child to the study team.	AEs/SAEs will only be collected for the child participant.

Summary of Key Changes from Version-01 to Version-02:

Affected Section(s)	Summary of Revisions Made from V-01 to V-02	Rationale
Title page, 10.1.6	DCOC/overall PI changed from Jessica Snowden, MD, to Song Ounpraseuth, PhD.	Changed due to departure of Dr. Snowden from UAMS/DCOC
1.1, Synopsis	Study Description section: Deleted "During the first 4 months of the school year," from the sentence that now reads, "Families with children aged 5-11 years with a diagnosis of ADHD who are initiating medication for the first time (either new diagnosis or prior diagnosis without stimulant treatment) will be approached to participate".	The study will no longer begin at the start of the school year due to delays in study initiation.
1.1, Synopsis	Description of Sites Enrolling Participants section: Added the paragraph, "The staff at the selected practices are not directly involved in the design, execution, or analysis of this research study and are thus considered not engaged in research activities. However, practice personnel may carry out specific, limited supportive tasks as directed by the site study team, provided these tasks remain appropriately constrained in scope."	Added to clarify role of practice staff at the sites, emphasizing that they are not undertaking research activities.

1.1, 2.2, 4.2, 5.1	"Rural practices" changed to "practices with high rural and/or underserved populations." Practices will be designated as underserved if at least 40% of its pediatric patient visits are covered by Medicaid.	Changed in response to available practices.
1.1, 4.1, 4.2, 4.3, 5.5.3, 6.1.1, 6.2, 6.3, 7.3	The schedule for receiving RAMP requests in the intervention group and digital education handouts in the control group changed from "weekly for 4 weeks, then monthly for 5 months" to "weekly for 4 weeks, then monthly for 2 months." The last check-in will be at 2 months instead of 4 months. The 180-day Vanderbilt study assessment was removed. The 90-day study assessment must be completed by day 105.	Study duration shortened from 6 months to 3 months.
1.1, 4.1, 6.2	Study duration shortened to 10 months from 14 months. Study start-up and site education shortened from 2 months to 1 month. Rolling recruitment and enrollment shortened from 4 months to 3 months. Participant activities changed from 10 months to 6 months during the study timeline. Provider participant activities shortened from 12 months to 7 months. Caregiver/child participant duration changed from 6 months to 3 months.	Timeframe for study shortened.
1.1, 5.1	"Two rural pediatric or family practice locations will be selected from each of two ISPCTN awardee sites, for a total of 4 practice locations," changed to, "A total of 4 practice locations will be selected from two ISPCTN awardee sites, with each site recruiting at least 1 practice location for participation."	Changed in response to available practices.
1.1, 5.1	Added that children who have not received stimulants for the prior 6 months will be eligible.	Participant eligibility expanded in response to available practices.
1.1, 3, 8.1, 9.4.3	Secondary endpoint changed from, "Proportion of available RAMP reports that are documented as reviewed by a provider," to, "Proportion of provider review surveys with at least one	There may not be a unique provider survey linked to each parent/teacher RAMP form returned. Provider surveys are

	available RAMP Report where review is documented."	linked to all returned parent/teacher RAMP forms for one study time point, for one child participant.
1.2.1, Schema (Caregiver/child Participants)	Schema illustrated timeframe shortened from 6 months to 3 months.	Timeframe for study shortened.
1.3 Schedule of Activities, Study Level Activities Table	Footnote added: The information gathered from chart review and RAMP reports will not exceed the presence/absence of the paper symptom form/RAMP report, the date/time they were submitted, and whether or not they were reviewed by a provider. Timeframes and scheduled activities adjusted to reflect shortened study timeframe	cIRB contingency & timeframe for study shortened and shifted due to delays in study initiation.
1.3 Schedule of Activities, Participant Activities Table	Footnote added: Each monthly period is defined as 28 days.	Clarification
2.2 Background	Added, "The program was created at the Medical University of South Carolina and replicated at University of Mississippi Medical Center. Neither institution has a financial interest in the system and there is no intention to commercialize."	cIRB contingency
2.3.1, 5.5.4	Providers will receive one reminder prompt at 24 hours if the RAMP report review has not been completed. Previously, two reminders were scheduled at 24 and 48 hours.	Change made to fit all RAMP requests, reminders and reviews into one week.
4.1 Overall Design	Enrollment will be tracked per practice (previously per site). Added opening enrollment as a possible response to multiple practices failing to meet enrollment goals.	Practices may face unique challenges.
5.5.1, Recruitment of Potential Participants	Replaced "from each practice location will occur during the enrollment period to review recruitment approaches, consider successes, barriers, and requirements to support ongoing enrollment, as needed" with "may occur during the enrollment period to keep practice staff informed about the progress of the study."	Added to clarify role of practice staff at the sites, emphasizing that they are not undertaking research activities.

5.5.3, Caregiver Participant Retention	Caregiver compensation changed from, "at enrollment ($100), after completing the mid-point study assessment ($50), and after completing the final study assessment ($50)" to "after completing the enrollment visit ($100), and after completing the 90-day study assessment ($100)."	Timeframe for study shortened.
5.5.4 Provider Participant Retention	Description of provider compensation "at enrollment," changed to, "after signing the provider consent form," to make it clear that this is for their own enrollment. Also clarified that, "Referring potential participants to the study team is a normal activity for providers in clinical trials, and providers who join the study will not receive extra payments for referrals. Providers will not join consent encounters for their patients. Given that providers typically enjoy salaries well above the Unites States median income, the compensation provided should not unduly influence their decision to participate."	cIRB contingency
6.1.1 Study Intervention Description	Research coordinator procedures: "monthly chart reviews" changed to "periodic chart reviews."	Changed to add flexibility.
6.1.1 Study Intervention Description	RAMP Reports completed by caregivers: "RAMP Reports will be able to be answered for 48 hours during the weekly period, and then for 1 week during the monthly period," changed to, "RAMP Reports will be submittable for 72 hours after initial release."	Change made to fit all RAMP requests, reminders and reviews into one week.
6.1.1 Study Intervention Description	Schema under RAMP Reports completed by teachers, starred text between arms: Parent changed to caregiver. Reminder schedule changed (corrected) from Tue/Wed to Mon/Tue.	Sunday to Tuesday is 48 hours, not 24 hours.
6.1.1 Study Intervention Description	Provider Review Report: Added, "While providers will have access to the platform at all times, there will be one provider survey to document review for any given week that there	Relates to change in secondary endpoint.

	is either a caregiver survey, teacher survey or both to review. This provider survey will be available only after the submission window for caregivers and teachers has ended." Also corrected that the provider survey contains only 2 questions.	
7.2, Participant Discontinuation/withdrawal from the Study	Streamlined language by deleting "by indicating to their health care provider, site champion or research coordinator that they wish to withdraw. Site champions will be identified to participants during the enrollment process." Also deleted "site champion" in last paragraph of section.	Clarification
8.3.1 Definition of Adverse Events (AE)	Changed "AEs associated with the study" to "AEs related to the study"	Clarification
8.3.4 Time Period and Frequency for Event Assessment and Follow-up	Replaced "Participants may report severe AEs for seven days after completing all study procedures and SAEs for 30 days after completing all study procedures, " with "Clinic providers participating in this study will be asked to remind caregiver participants to report any medication side effects or events that caused them to seek medical assistance or hospitalization to the study team."	Clarification
8.3.9 Reporting of Pregnancy	Added "the child" so that sentence reads "Given the age of the child participants . . ." Added the sentence, "Child participants' pregnancies will be reported in the EDC but not followed for the pregnancy duration."	Clarification
10.1.1.2 Consent Procedures and Documentation	Clarified that the study team will return "a copy of" the countersigned consent form to participants.	Clarification
10.1.6, Key Roles and Governance	Added DCOC Co-PIs Fred Pryor, PhD, and Sherry Courtney, MD.	DCOC staff change
10.1.12 Publication and Data Sharing Policy	Changed contact person from Jeannette Lee to Song Ounprasueth.	DCOC staff change

Table of Contents

Statement of Compliance ... 12
1 Protocol Summary ... 13
1.1 Synopsis ... 13
1.2.1 Schema (caregiver/child Participants) ... 16
1.2.2 Schema (study timeline, study team activities and provider activities) ... 17
1.3 Schedule of Activities (SoA) ... 18
2 Introduction ... 20
2.1 Study Rationale ... 20
2.2 Background ... 21
2.3 Risk/Benefit Assessment ... 23
2.3.1 Known Potential Risks ... 23
2.3.2 Known Potential Benefits ... 23
2.3.3 Assessment of Potential Risks and Benefits ... 23
3 Objectives and Endpoints ... 24
4 Study Design ... 25
4.1 Overall Design ... 25
4.2 Scientific Rationale for Study Design ... 26
4.3 End of Study Definition ... 27
5 Study Population ... 28
5.1 Inclusion Criteria ... 28
5.2 Exclusion Criteria ... 29
5.3 Lifestyle Considerations ... 30
5.4 Screen Failures ... 30
5.5 Strategies for Recruitment and Retention ... 30
5.5.1 Recruitment of Potential Participants ... 30
5.5.2 Screening and Enrollment Procedures ... 31
5.5.3 Caregiver Participant Retention ... 31
5.5.4 Provider Participant Retention ... 32
6 Study Intervention ... 33
6.1 Study Intervention(s) Administration ... 33

6.1.1 Study Intervention Description 33
6.2 Measures to Minimize Bias: Randomization and Blinding 35
6.3 Study Intervention Compliance 35
7 Study Intervention Discontinuation and Participant Discontinuation/Withdrawal 37
7.1 Discontinuation of Study Intervention 37
7.2 Participant Discontinuation/Withdrawal from the Study 37
7.3 Lost to Follow-Up 37
8 Study Assessments and Procedures 39
8.1 Efficacy Assessments 39
8.2 Safety and Other Assessments 39
8.3 Adverse Events and Serious Adverse Events 39
8.3.1 Definition of Adverse Events (AE) 39
8.3.2 Definition of Serious Adverse Events (SAE) 40
8.3.3 Classification of an Adverse Event 40
8.3.4 Time Period and Frequency for Event Assessment and Follow-Up 41
8.3.5 Adverse Event Reporting 41
8.3.6 Serious Adverse Event Reporting 42
8.3.7 Reporting Events to Participants 43
8.3.8 Events of Special Interest 43
8.3.9 Reporting of Pregnancy 43
8.4 Unanticipated Problems 43
8.4.1 Definition of Unanticipated Problems (UP) 43
8.4.2 Unanticipated Problem Reporting 44
8.4.3 Reporting Unanticipated Problems to Participants 44
9 Statistical Considerations 45
9.1 Statistical Hypotheses 45
9.2 Sample Size Determination 45
9.3 Populations for Analyses 45
9.4 Statistical Analyses 46
9.4.1 General Approach 46
9.4.2 Analysis of the Primary Endpoint(s) 46

9.4.3 Analysis of the Secondary Endpoint(s) 46
9.4.4 Safety Analyses 46
9.4.5 Baseline Descriptive Statistics 47
9.4.6 Planned Interim Analyses 47
9.4.7 Sub-Group Analyses 47
9.4.8 Tabulation of Individual Participant Data 47
9.4.9 Exploratory Analyses 47
10 Supporting Documentation and Operational Considerations 48
10.1 Regulatory, Ethical, and Study Oversight Considerations 48
10.1.1 Informed Consent Process 48
10.1.2 Study Discontinuation and Closure 51
10.1.3 Confidentiality and Privacy 51
10.1.4 Multi-site Communications (IRB-related) 52
10.1.5 Future Use of Stored Specimens and Data 52
10.1.6 Key Roles and Study Governance 53
10.1.7 Safety Oversight 54
10.1.8 Clinical Monitoring 54
10.1.9 Quality Assurance and Quality Control 55
10.1.10 Data Handling and Record Keeping 55
10.1.11 Protocol Deviations 56
10.1.12 Publication and Data Sharing Policy 57
10.1.13 Conflict of Interest Policy 57
10.2 Table and Figure Key 57
10.3 Abbreviations 58
11 References 60

STATEMENT OF COMPLIANCE

The trial will be carried out in accordance with International Conference on Harmonisation Good Clinical Practice (ICH GCP) and the following:

- United States (US) Code of Federal Regulations (CFR) applicable to clinical studies that are not regulated by the FDA, specifically, (45 CFR Part 46)

National Institutes of Health (NIH)-funded investigators and clinical trial site staff who are responsible for the conduct, management, or oversight of NIH-funded clinical trials have completed Human Subjects Protection and ICH GCP Training.

The protocol, informed consent form(s), recruitment materials, and all participant materials will be submitted to the Institutional Review Board (IRB) for review and approval. Approval of both the protocol and the consent form (including HIPAA authorization) must be obtained before any participant is enrolled. Any amendment or modification to the protocol will require review and approval by the IRB before the changes are implemented to the study. In addition, all changes to the consent form and HIPAA authorization will be IRB-approved. A determination will be made regarding whether a new consent needs to be obtained from participants who provided consent, using a previously approved consent form.

1 PROTOCOL SUMMARY

1.1 SYNOPSIS

Title: A Pilot Study of a Remote Attention-Deficit/Hyperactivity Disorder Monitoring Program (RAMP) for Children in Rural Areas

Study Description: This is a pilot study examining the use of an Attention-Deficit/Hyperactivity Disorder (ADHD) Monitoring Program during the early stages of treating ADHD in children living in rural settings. The Remote ADHD Monitoring Program (RAMP) will include provider ADHD Best Practice education, caregiver and teacher prompts for frequent RAMP Reports of inattention and hyperactivity symptoms and a provider dashboard with enrolled children. The RAMP Report includes the publicly available Vanderbilt Rating Scales (henceforth shortened to Vanderbilt Assessment), which is a commonly used, guideline-recommended tool for both diagnosis and management of ADHD for use by parents and teachers.[1] See Appendix 1. This assessment was initially validated in a referral population with teacher reporters in 1998 and with parent reporters in 2003, and subsequently in a community population with both reporters in 2013.[1-4] Additionally, school performance, medication side effects and behavioral therapy engagement will be solicited on a recurring basis. The results of this pilot will be used to design a full-scale efficacy trial in additional rural sites to expand diversity and assess generalizability.

> Remote ADHD Monitoring Program (RAMP) Report includes:
> - Inattention and hyperactivity questions from Vanderbilt assessment (18 total)
> - Medication side effects query
> - School performance query
> - Behavior health provider visits query

For this pilot study, children diagnosed with ADHD starting stimulant treatment for the first time in rural settings will be the focus of efforts to improve time to optimal symptom control, in an effort to maximize the treatment benefit for the academic year. We anticipate that more frequent provision of information by families and teachers to medical providers, enabled by technology and remote monitoring tools, will encourage more rapid improvement in symptom burden. This would be achieved through timely, even weekly, titration of medication per the current treatment guidelines, which is only possible with symptom reports.

The goals of this pilot study are 2-fold:

- Evaluate caregiver and teacher utilization of the RAMP platform early in the treatment course of ADHD
- Evaluate provider utilization of the RAMP platform in everyday practice and management of children early in the treatment course of ADHD

Participants will be recruited from four practices with high rural and/or underserved populations from two ISPCTN awardee sites. Participation will be required by all providers who manage ADHD at the four practices. Prior to the start of caregiver/child recruitment, all providers at the clinics will receive ADHD Best Practice education consistent with the 2019 American Academy of Pediatrics (AAP) clinical practice guidelines for diagnosis and management of children with ADHD.[5] Families with children aged 5-11 years with a diagnosis of ADHD who are initiating medication for the first time (either new diagnosis or prior diagnosis

without stimulant treatment) or have not received stimulants for the last 3 months will be approached to participate. Families will be approached until 36 caregiver/child dyads are enrolled (only one child in a family may be enrolled). Those enrolled will be randomized 1:1 to either the intervention group or control group. The intervention group will be enrolled in the RAMP, with prompts to provide frequent reports including ADHD symptoms. The control group will provide ADHD symptom reports through paper forms (usual care), on a provider determined schedule. While we will recommend that a Vanderbilt Assessment be used, providers may use a symptom screener of their choice for usual care. The control group will also receive digital education handouts about age-appropriate pediatric health topics (e.g., general health, exercise, digital media, sleep) in children on the same schedule as the RAMP requests are sent to caregivers and teachers for participants in the intervention group. Each group will have provider-directed medication management of ADHD (usual care).

The outcomes examined in the pilot include feasibility and acceptability of the RAMP to caregivers, teachers and providers. Participant utilization of the RAMP will be measured. Provider engagement with the RAMP will be explored using data review logs.

Objectives:

Primary Objective: **Evaluate completion of clinical assessments by caregivers and teachers.**

Aim 1. Evaluate the number of clinical assessments (paper assessments in control and RAMP Reports in intervention) per participant that are completed by caregivers and teachers and returned to providers for ADHD management.

Secondary Objective: **Evaluate provider acceptability of the RAMP through utilization measures.**

Aim 2. Evaluate the proportion of all submitted RAMP Reports that are reviewed by providers.

Endpoints:

Primary Endpoint: Number of completed caregiver and teacher clinical assessments (RAMP Report or paper assessment) that are returned to provider

Secondary Endpoint: Proportion of provider review surveys with at least one available RAMP Report where review is documented.

Study Population: Participants will include the dyads of children aged 5-11 years with stimulant-naïve diagnosis of ADHD (can be new diagnosis or prior diagnosis without stimulant treatment) or have not received stimulants for the last 3 months and their caregivers. They must attend one of the participating practices and be starting on stimulant medication for treatment of ADHD. The providers of the participating practices will also be considered participants for the secondary objective.

Phase: Pilot

Description of Sites Enrolling Participants: A total of 4 practice locations will be selected from two ISPCTN awardee sites, with each site recruiting at least 1 practice location for participation. Practices should care for at least 700 patients aged 5-11 years and should regularly diagnose and manage pediatric ADHD. Practices

will be designated as rural if they are at least 30 minutes travel distance from a pediatric academic medical center (similar to prior studies) and are located in a city with fewer than 50,000 people.[6-8] Practices will be designated as underserved if at least 40% of its pediatric patient visits are covered by Medicaid.

The staff at the selected practices are not directly involved in the design, execution, or analysis of this research study and are thus considered not engaged in research activities. However, practice personnel may carry out specific, limited supportive tasks as directed by the site study team, provided these tasks remain appropriately constrained in scope.

Description of Study Intervention: The intervention group will be enrolled in the RAMP. The RAMP platform used for this study will be an adaptation of an existing program and will be housed in REDCap, a HIPAA-compliant data collection tool. [9,10] Caregivers and teachers will receive text-based requests for reports with embedded links to select radio button questionnaires directly on their mobile device (without any login requirement). Questions include current inattention/hyperactivity symptoms, school performance, and any engagement in behavioral therapy. These areas of information are typically considered by providers to aid in decision making regarding medication dose adjustments. Inattention/hyperactivity symptom questions and scoring is taken directly from the Vanderbilt Assessment. These requests for information will be distributed weekly for 4 weeks, then monthly for 2 months.

Providers will receive ADHD Best Practices education at the start of the study including recommended use of the Vanderbilt Assessment and appropriate follow-up as well as how to utilize the RAMP platform. The project is not focused on the effect of provider training, as these are not new guidelines, but rather addresses other potential barriers that are unique to rural settings. After enrollment of caregiver/child participant dyads, providers will be alerted by their preferred means (text or email) when new RAMP Reports are available for review. Following RAMP report review, the provider will answer a brief 2-question, survey regarding their response to the report. Providers will be able to view the individual as well as prior reports in their dashboard view.

Research coordinators will monitor caregiver and teacher activity, including providing RAMP Report responses and provider activity in reviewing responses, and following up with caregivers and providers as outlined in the retention plan to determine if there have been gaps in or a lack of engagement.

The control group caregiver participants will receive digital education handouts about age-appropriate general pediatric health topics (e.g., exercise, digital media, sleep) weekly for 4 weeks then monthly for 2 months (the same interval as RAMP requests in the intervention group).

- Clinical assessment: any paper assessment or RAMP Report reviewed by health care provider
- Study assessment: Vanderbilt Assessment at Day 90, reviewed only by study team

- The Diagnostic assessment (time 0) is the only document that fall into both categories.

All caregiver participants will receive a Vanderbilt Assessments as study assessment at day 90 (either paper or digital, based on preference solicited at enrollment). They will only be reviewed by the study team (and not any health care provider) and will clearly be marked as such. These study assessments will serve as markers of retention.

Study Duration: 17 months – 1 months study start-up and local site provider ADHD Best Practice education, 10 months rolling recruitment and enrollment to reach 36 total caregiver/child dyads with a 3-month enrollment pause from May 6, 2025 through July 31, 2025, while children are out of school. Based on timing of enrollment there will be up to 14 months of provider participant activities and 13 months of caregiver/child participant activities at the study level.

Participant Duration: 3 months

1.2.1 SCHEMA (CAREGIVER/CHILD PARTICIPANTS)

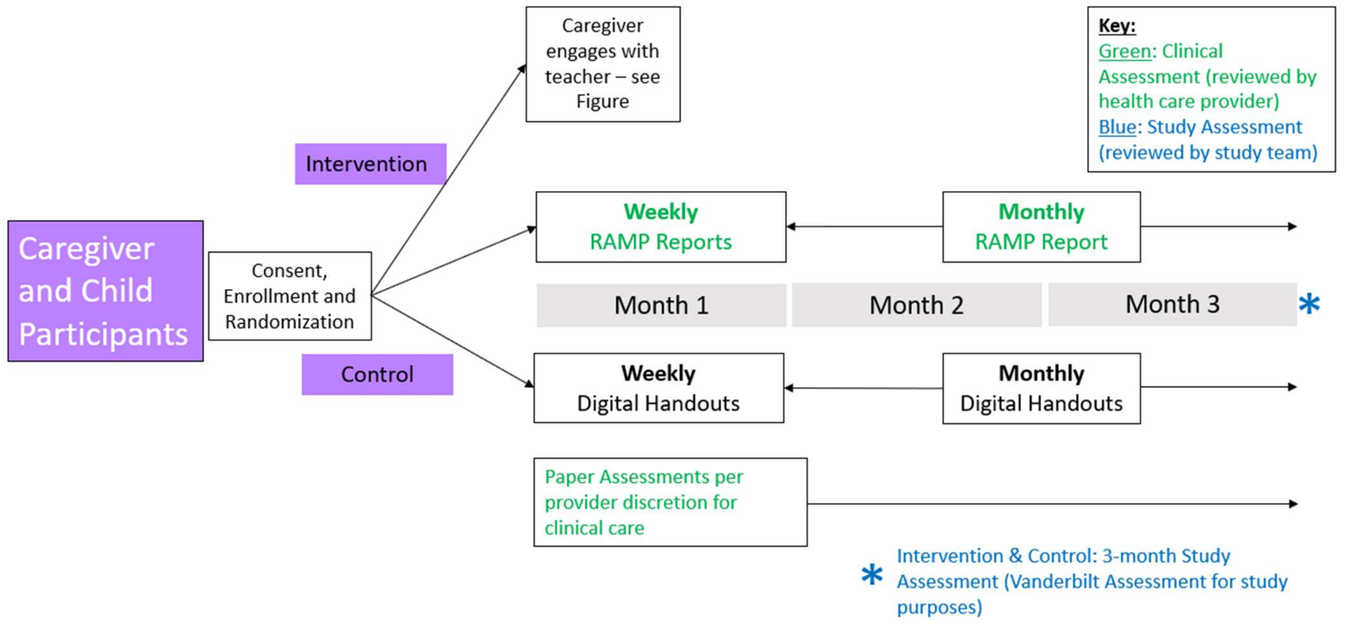

1.2.2 SCHEMA (STUDY TIMELINE, STUDY TEAM ACTIVITIES AND PROVIDER ACTIVITIES)

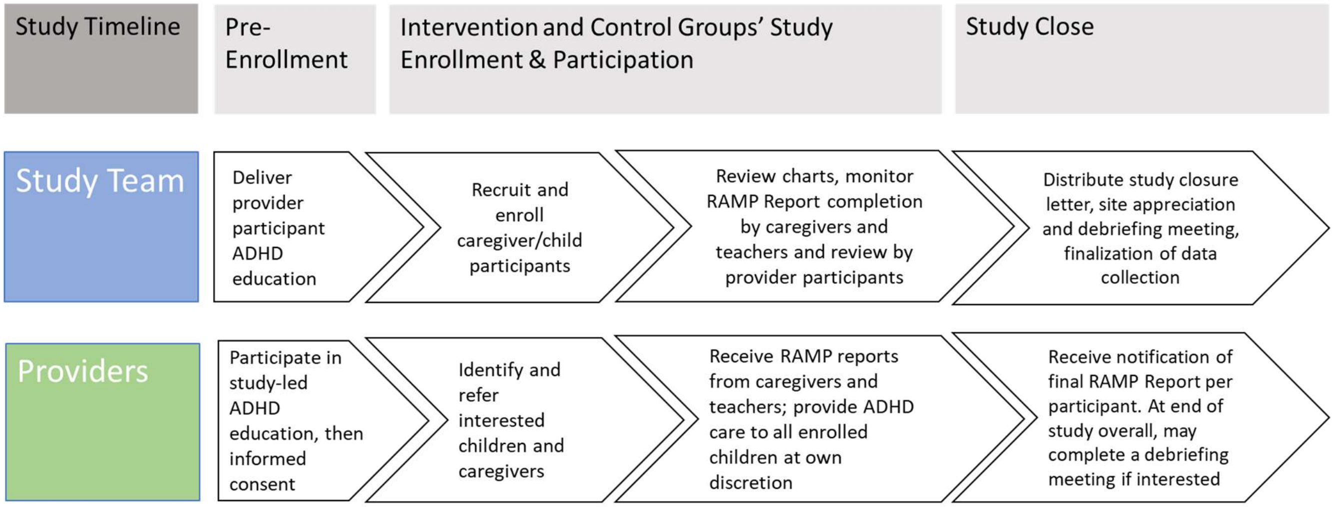

1.3 SCHEDULE OF ACTIVITIES (SOA)

	Anticipated Study Period (in Months)								
**Study Level Activities**	**1**	**2**	**Months 3 - 11**	**12**	**13**	**14**	**15**	**16**	**17**
Provider ADHD and study education	X								
Provider consent	X	X							
Caregiver/child enrollment, consent, and randomization		X	X**						
Participant on-study activity		X	X	X	X	X			
Chart and RAMP review and abstraction* by research coordinator		X	X	X	X	X			
Analysis and write-up							X	X	X

*The information gathered from chart review and RAMP reports will not exceed the presence/absence of the paper symptom form/RAMP report, the date/time they were submitted, and whether or not they were reviewed by a provider.

**Enrollment will pause for 3 months during the summer months when children are out of school.

Study Activities	Caregiver/Child Dyads	Teachers	Providers
Informed consent	X		X
Randomization	X		
Complete RAMP reports (intervention arm only)	X	X	
Receive digital handouts (control arm only)	X		
Allow access to clinical baseline assessment and complete study assessments	X		

RAMP platform orientation			X
Best practice education			X
Review RAMP reports			X
Complete Provider Survey			X
Receive Compensation	X		X

**Participant Activities**	**Day -35—Day 0**	**Day 0**	**Day 1-28**	**Day 29-90**	**Day 90 (-30/+15)****
Pre-study visit with provider* (ADHD diagnosis made, stimulant started)	X				
Research coordinator contacts and eligibility screen, principal investigator review	X				
Enrollment and informed consent		X			
Randomized		X			
Diagnostic Assessment requested from provider		X			
RAMP Report request (caregiver and teacher) for intervention group only			Weekly	Monthly***	
Digital education handout (control group only)			Weekly	Monthly***	
Study Assessments (not used in clinical management; retention measure)					X

*Caregiver/child participant dyads will be enrolled within 5 weeks (35 days) of the child being prescribed stimulants.
**The window for the 90-day Study Assessment is -30 days to +15 days. Study Assessments must be received by the study team within this window.
***Each monthly period is defined as 28 days.

2 INTRODUCTION

2.1 STUDY RATIONALE

Children with ADHD often do not receive evidence-based guideline-driven care, particularly those living in rural areas. Children in rural settings are less likely to receive an ADHD diagnosis, receive timely follow-up and are less likely to be prescribed stimulant medications than their urban counterparts.[11,12] Challenges arise from both patient-level and provider-level barriers. Patient-level barriers include less access to pediatric behavioral health care, higher rates of chronic conditions, and overall poorer health status.[13] Providers in rural areas experience unique challenges, related to practice setting, to using these portals including variable electronic medical records (EMR)s and level of technology support.[14] Additionally, providers in rural environments must incorporate the patient-level reality of distance to care when developing management plans.

System-level challenges include the multiple steps needed to make the ADHD diagnosis starting with recognition of behavior or academic challenges, followed by repeated cycles of gathering information from teachers and caregivers, frequent close follow-up and medication titration. These steps require a high degree of caregiver engagement because the cycles needed for appropriate medication titration are caregiver-driven. In a rural environment, disruptions of these cycles may be amplified due to distance and access barriers. **Communication tools that use digital platforms to facilitate caregiver engagement with these titration cycles are not widely used.** Successful communication and engagement early in the diagnostic process may lead to faster symptom control which may positively reinforce future engagement.

Successful solutions for increasing caregiver engagement in urban areas, using a single EMR or first-generation portal, showed improved communication between caregivers, providers and teachers, but have not been tested in rural environments.[15,16] Traditional care models do not address the unique challenges of rural health, leading to disparities in outcomes. With the explosion of virtual care during the COVID-19 pandemic, innovative approaches using patient-preferred text-based platforms may be more responsive to the unique needs of rural children with ADHD. **A telehealth ADHD care model tailored to rural primary care settings could ultimately enable improved adherence to ADHD treatment guidelines.**

The need for improving ADHD care is universal; however, solutions that have worked in urban environments have not been evaluated in rural context with the aforementioned patient and provider barriers. Previous solutions for chronic conditions that have worked in urban areas have not necessarily translated well to rural or underserved settings.[17-19] This will be one of the first studies to evaluate the feasibility of structured remote symptom monitoring in rural settings using the validated Vanderbilt Assessment in children with ADHD, including its ability to engage patients, providers and teachers. We will work with large rural practices (caring for >700 children in the study inclusion age group) in small cities (<50,000 people) that experience shortages of pediatric sub-specialists (e.g., developmental-behavioral pediatricians) and mental health providers due to geographic barriers (> 30 minutes travel distance from a pediatric academic medical center). With a pediatric ADHD prevalence of 11% and 700 children in the study inclusion age group, practices will have on average 77 children with ADHD across the inclusion age group. Many of these children will be initiating stimulant

medication for the first time considering the median age of onset is 6 years.[20] Use of the guidelines to manage symptoms is linked to improved educational and social outcomes. Symptom control reduces the rates of depression, anxiety, substance use disorder, and other health impairments that can persist into adulthood.[21-23] **This proposal will lay the groundwork for innovative use of technology to improve early outcomes for children with ADHD in rural communities.**

2.2 BACKGROUND

Following evidence-based guideline recommendations for ADHD, including timely titration, use of structured reports and appropriate follow-up, may improve care. The 2019 AAP Clinical Practice Guidelines for the management of ADHD state that stimulant medications can be effectively titrated on a 7-day basis.[5] Additionally, the AAP recommends that symptoms and impairment should be documented through structured reports.[5] Adhering to follow-up recommendations and higher numbers of structured reports from teachers in the first year of treatment has been shown to significantly predict greater decreases in patient total symptom scores.[24] Similarly, the Multimodal Treatment Study of Children With ADHD found that rapidly titrated medication dosing and close communication between the clinician and the child's teacher yielded improved outcomes over usual care.[25]

With the steadily progressive nature of academic activity and social skills expected during the elementary school years, any delays in improving symptoms will potentially have substantial negative long-term impact. The recommendations to advance stimulant therapy weekly, once the decision has been made to initiate it, are to support the most rapid, safe improvement in symptoms in order to optimize academic and social development opportunities. Delays in symptom control, once a diagnosis has been made, only serve to delay improvements in educational and social functioning. However, once optimal symptom control is achieved, children are still delayed in attaining milestones and may remain educationally and socially delayed even as they make improvement. As these educational and social skills are continuously progressing during these years, delays in improved functioning have long-term impact.

A frequently used method of gathering symptom information from different settings is the Vanderbilt Assessment.[26] The Vanderbilt Assessment is a well-validated instrument that includes 18 questions on a 0-3 Likert scale regarding inattention and hyperactivity, as well as additional sections for screening for anxiety and depression.[3] Versions are available for parent and teacher completion, for use during the diagnostic phase and subsequent management of ADHD. It is widely used as it is free of charge to providers and easy to score. (Appendix 1). Caregivers typically complete paper versions. This can be done in the office or at home, and returned at the subsequent in-office visit to the provider. In some cases, the teacher version is given to the caregiver to give to the child's primary teacher. The teacher completes the assessment, returns it to the caregiver (typically through the child's backpack), and the caregiver returns it to the provider at the next appointment. Alternatively, some practices allow teachers to fax it directly back to the practice, for matching in the office to the appropriate child. This cycle should repeat until the child's symptoms reach a substantially improved level. With the multitude of steps involved in this process, it is no surprise that the use of structured reports at follow-up is only 10%.[27]

Unfortunately, physicians encounter challenges in following these ADHD guidelines, particularly obtaining structured reports of symptoms (such as Vanderbilt Assessments), optimizing the timing of visits, and titrating quickly to optimal symptom control.[5] Furthermore, children with ADHD in rural areas encounter specific challenges to receiving this guideline-recommended care. They are more likely to suffer from challenges with access to primary care and specialty care, including behavioral health.[28,29] Children in rural areas are less likely to receive the diagnosis of ADHD or stimulant treatment than those in urban areas.[12] In the United States, primary care providers such as pediatricians and family medicine providers provide most ADHD care.[30] Despite clear recommendations to use structured reports such as a Vanderbilt Assessment at diagnosis and follow-up, Epstein *et al.* found that the use of structured reports at the time of diagnosis in community practices was around 50% and use at follow-up was as low as 10%.[27] As we review the traditional steps in obtaining these reports from caregivers and teachers, it is clear that there is an important unmet gap for children in rural settings, that if addressed, could substantially impact outcomes.

Remote patient monitoring is a form of telehealth that involves the collection and transmission of health data from a patient to their health care team using digital health technologies.[31] It has been shown to be successful in a wide variety of pediatric conditions, including those gathering subjective data (such as pain) as well as objective data (such as glucose).[32,33] While remote monitoring of ADHD is not well-studied, the use of electronic tools such as portals have been shown to improve some ADHD-care quality measures and resulted in improved outcomes including reductions in parent-rated ADHD symptoms as compared to usual care in urban settings.[16,34] The use of these EMR-embedded tools has been studied in large networks with single-EMR clinical systems, and non-EMR-based portals have been studied in urban and suburban community practices that were linked. Providers in rural practices may encounter challenges with EMR-embedded tools because they have varied, office-based, electronic medical record vendors with less pediatric focus due to practice size. Additionally, providers in rural practices may lack the information technology (IT) infrastructure associated with large academic centers to create pediatric disease-specific tools.

The need for improving ADHD care is universal; however, the translation of solutions that have worked in urban environments is not known for patients in rural environments. Delivery of health services to pediatric patient homes using variable modalities of telehealth including remote patient monitoring has been widely recommended across various disease states.[35,36] The RAMP will be a non-EMR based program that delivers text-message RAMP Report requests to caregivers and teachers and compiles patient data in provider dashboards. It eliminates the multiple handoffs of paper symptom assessment forms between caregivers, teachers and providers. Data is managed in Research Electronic Data Capture (REDCap), a secure web platform for building and managing online databases and surveys. The RAMP is being adapted from an existing program used successfully for behavioral health screening for pregnant women. The program was created at the Medical University of South Carolina and replicated at University of Mississippi Medical Center. Neither institution has a financial interest in the system and there is no intention to commercialize. This program has screened close to 400 women and has shown superior clinical outcomes compared to usual care.[10] A non-EMR based platform will allow for evaluation of this approach across rural sites, without requiring significant IT investments, and eliminating the impact of the variability of EMRs across rural practices. Finally, a text-based platform to deliver

asynchronous care is in line with the most current user preferences for phone-based services, representing next-generation patient engagement.

The research questions for the pilot project are as follows: First, is the use of RAMP acceptable to caregivers and teachers living in rural communities? Second, will providers of ADHD care in rural and underserved communities engage with dashboards collecting the RAMP Reports from caregivers and teachers?

2.3 RISK/BENEFIT ASSESSMENT

2.3.1 KNOWN POTENTIAL RISKS

The study poses minimal risk to caregiver/child dyads. These dyads will provide protected health information (PHI) as well as ADHD symptoms. The RAMP will be housed on a secure platform and sites will use secure email for written communication between participants when possible. While the study team will make every effort to store this electronic information in secure electronic databases, there remains a risk of accidental as well as mandated disclosure of PHI.

A secondary risk to participants would be if challenges are encountered while navigating a new technology in an untested environment. There is a risk that a caregiver could provide information that does not get reviewed in a timely fashion by a provider. To mitigate this, caregivers are instructed in the platform to reach out to their provider directly if there is a serious concern. This risk to participants is no greater than what would be incurred during routine ADHD management. Additionally, if a provider does not review a report, he or she will receive a reminder prompt after 24. There may be a delay in response to participants if elements of the technology are not clear. An orientation to the platform will be conducted at the start of the study with all providers that may interact with the platform. If there are any challenges encountered during the process, a member of the study team will be readily available to assist.

2.3.2 KNOWN POTENTIAL BENEFITS

Previous studies of similar programs have shown benefit to participants and providers but not in rural settings. If the intervention is successful, caregiver and child participants may experience a more rapid titration of medication to optimal dosing, increased communication with health care providers as well as with teachers, and faster referrals to additional services.

The control group will receive digital education handouts about age-appropriate pediatric health topics through email.

2.3.3 ASSESSMENT OF POTENTIAL RISKS AND BENEFITS

We do not anticipate significant health risks to participants and will make every effort to minimize the possible risks described in Section 2.3.1. The benefit of understanding the effect of a Remote ADHD Monitoring Program on improving care for children in rural areas outweighs the risks. Standards of medical care provided to child participants will not be altered based on participants' study-related activities.

3 OBJECTIVES AND ENDPOINTS

Objective	Endpoint	Justification
Primary		
**Evaluate completion of clinical assessments by caregivers and teachers.**	Number of completed caregiver and teacher clinical assessments (RAMP Report or paper assessment) that are returned to provider	The level of ongoing caregiver and teacher engagement in each arm will be important for planning a full-scale trial. Absolute number will be used as this is what has been shown to improve ADHD symptom control.[24] Increasing communication between caregivers and providers is important in enhancing the care of individuals with ADHD and literature in this area in rural settings is lacking.
Secondary		
**Evaluate provider acceptability of the RAMP through utilization measures.**	Proportion of provider review surveys with at least one available RAMP Report where review is documented	Because improvement in care requires bi-directional communication, the review of newly available reports by the provider will be important to measure. While we anticipate that providers will find accessing this information easy and helpful, it may be difficult to incorporate into usual workflow.

4 STUDY DESIGN

4.1 OVERALL DESIGN

The design is a pilot study that tests the utilization of an ADHD monitoring platform in a rural setting. We will examine utilization from the caregiver, provider, and teacher perspectives. We hypothesize that this platform will facilitate information provision from families and teachers to health care providers and will be a useful and acceptable means of ADHD symptom monitoring.

The entire timeline will span 17 months with three phases. In the first phase of the study, the providers from the participating practices will receive ADHD Best Practices education and training in the portal use, and will be asked to provide informed consent. A member of the clinic staff (nurse, medical assistant or practice manager) will be identified as site champion. The site champion will facilitate communication between the site study team and the clinic staff. The second phase of the study will focus on child/caregiver and provider utilization. Providers will identify patients with a diagnosis of ADHD who are being started on stimulant medication to be contacted by study staff for enrollment. Research coordinators will discuss the study, perform eligibility screening and obtain consent (if deemed eligible by PI). The consented caregiver/child dyads will be randomized to intervention (requests to submit the RAMP Reports for 3 months) or control (usual care with attention control) groups. Families will be approached until 36 caregiver/child dyads are enrolled (only one child in a family may be enrolled). Each practice will have 6 weeks to enroll 9 participants. After the 6 weeks, practices with low enrollment will receive additional supports. Each week, progress to target will be evaluated to determine if any practice needs additional resources. If multiple practices do not meet enrollment goals, the study team may consider relaxing exclusion/widening inclusion criteria (ex. include patients undergoing re-titration, widen age criteria) and/or opening enrollment. Enrollment will be paused for 3 months from the last month of school to the start of the new school year (beginning of May to end of July). This will decrease the burden on practices and study staff during the time of the year with the fewest number of ADHD diagnoses. Once enrolled, caregiver/child participants will be randomized to either the intervention or control group and followed for approximately 3 months. Participants randomized to the intervention group will submit RAMP reports weekly for 4 weeks and then monthly for 2 months. Participants randomized to the control group will receive digital education handouts at the same frequency as the RAMP reports in the intervention arm. Research coordinators will periodically review the child participant's EMR and mark how many paper symptom assessments are returned while enrolled on the study for both groups. Research coordinators will also access the RAMP platform to log RAMP reports returned by caregivers/teachers, RAMP reports reviewed by providers, and provider survey results. All caregiver participants will complete a 90-day Vanderbilt Assessment as a retention measure (see section 1.2.1 for more details). This phase will continue until all 36 participants have completed their 3-month study period. In the third phase, the above endpoints will be calculated, the full-scale trial will be designed, and the findings presented in a manuscript.

4.2 SCIENTIFIC RATIONALE FOR STUDY DESIGN

Rationale for site selection: The overall goal of this project is to improve the care of children in rural and underserved populations with ADHD. We are using strategies that have been proven successful in urban areas and refining and testing these in rural areas. There is a documented need to address the deficit in care caused by geographic distance and variability in electronic medical records (EMRs) across rural environments. In order to successfully implement the intervention while still targeting a rural population, we will seek large rural practices (caring for >700 children in the study inclusion age group) in small cities (<50,000 people) that experience shortages of pediatric sub-specialists (e.g., developmental-behavioral pediatricians) and mental health providers due to geographic barriers (>30 minutes travel distance from a pediatric academic medical center). With 700 children and a pediatric ADHD prevalence of 11%, we would expect on average 77 children with ADHD across the inclusion age group. Many will be initiating stimulant medication for the first time considering median age of onset is 6 years.[20] A driving distance of 30 minutes was used as it is a standard that has been proposed by health departments and used in prior studies.[6,7,37]

Rationale for caregiver/child dyad selection: We will select children 5-11 years of age with a diagnosis of ADHD without serious mental health comorbidity, who have not previously been treated with a stimulant medication or have not received stimulant medications for the last 3 months. This includes a new diagnosis of ADHD or a prior diagnosis without stimulant treatment. Children aged 5-11 years are most commonly newly diagnosed with ADHD and the caregiver report of symptoms in this age group is of utmost importance.[20,38] We seek children with a stimulant-naïve ADHD diagnosis because they need to have medications titrated quickly in order to optimize symptom control and support improved academic and social outcomes. Lastly, we will exclude children with serious mental health or neurodevelopment conditions as their care may be more complicated, necessitating in-person visits.

Rationale for randomizing at the patient level: RAMP is an automated communication tool designed to eliminate the work burden for providers of requesting and receiving ADHD symptom data from participants. Thus, providers will not be able to reproduce the RAMP intervention for control participants. However, this is an unblinded pilot, and we do not expect providers to change their patient counseling based on participant condition allocation. Therefore, providers should not be confused by receiving RAMP Reports for some study participants (in the experimental condition) and not receiving RAMP Reports for others (in the control). While providers may increase the frequency of their self-initiated outreach to control participants because of the frequency with which they receive RAMP Reports for experimental condition participants, this bias would likely result in more Type II (false-negative) errors for determining intervention effectiveness, which is preferable to Type I (false-positive) errors.

Rationale for using digital education handouts: The digital education handouts will be sent via email to the control group and will serve as an attention control. They will consist of information regarding a general pediatric health topic and will be emailed at intervals matching the RAMP requests in the intervention group.

Rationale for the schedule of study assessments: The study team will collect the diagnostic assessment from both the intervention and control groups at 0 months (enrollment), as well as a study assessment (not shared

with provider and clearly marked as such) at Day 90 (-30/+15) –– the study final participant follow-up. The diagnostic assessments will be on paper and will be obtained from the diagnosing provider with caregiver permission. The subsequent study assessments will be obtained either on paper or electronically (caregiver choice). These study assessments will serve as markers of retention. Caregiver participant incentives will be based on return of these study assessments.

4.3 END OF STUDY DEFINITION

The end of the study is defined as the date when all study activities for all participants are completed or determined to be out of window as defined in this protocol.

The study will end for caregiver/child participants after they have reached 90 days post enrollment and either completed their final study assessment or failed to complete it by Day 105.

The study will end for provider participants after they have completed the provider survey linked to their last enrolled patient's 3-month RAMP report, or failed to complete it within the review window.

5 STUDY POPULATION

5.1 INCLUSION CRITERIA

Caregiver/child dyad eligibility: To be eligible to participate in the trial, caregiver/child dyads must attend one of the four participating primary care clinics and meet the following inclusion criteria:

The child must

- be under the care of a participating provider
- be aged 5-11 at enrollment
- have a diagnosis of ADHD
- be initiating stimulant medication for treatment for the first time or have not received stimulant medication for the last 3 months
- be attending in-person elementary school

Primary caregiver must

- be willing and legally able to give consent
- have access to a smartphone
- be English-speaking
- reside with the child at least 3 days per week
- complete an initial symptom assessment prior to starting stimulant treatment and be willing to provide a copy to the study team

Eligible children include those receiving a new ADHD diagnosis, those having a prior diagnosis without stimulant medication treatment, and those having a prior diagnosis but have not received stimulant medication for the last 3 months. Children only receiving adjunct (non-stimulant) medication are ineligible. Only one child per household can enroll. The participating caregiver is defined as the parent or legally authorized representative (LAR) who can consent for their child's medical treatment and should be with the child at least 3 days per week.

Practice selection: A total of 4 pediatric or family practice locations will be selected from two ISPCTN awardee sites, with each site recruiting at least 1 practice location for participation. Practices should care for at least 700 patients aged 5-11 years and should regularly diagnose and manage pediatric ADHD. With 700 children and a pediatric ADHD prevalence of 11%, we expect on average 77 children with ADHD across the inclusion age group, many of whom will be initiating stimulant medication for the first time, considering median age of onset is 6 years.[20] Practices will be designated as rural if they are at least 30 minutes travel distance from a pediatric academic medical center (similar to prior studies) and are located in a city with less than 50,000 people.[6-8] Practices will be designated underserved if at least 40% of its pediatric patient visits are covered by Medicaid. Practices must use an EMR as this pilot aims to use typical practices in preparation of the full-scale trial. 2019 National Electronic Health Records Survey found that 89.9% of office-based physicians were using an EMR.[39]

Use of electronic prescribing is preferred but not required. Practices must also be willing (and preferably experienced) in using the Vanderbilt Assessment as their primary tool for ADHD diagnosis and management. Providers must agree in concept to participate in the ADHD Best Practice education, as well as instruction on using the RAMP platform and will provide informed consent (see provider eligibility below). Interested ISPCTN sites will assess eligible practices and choose the best fit. Locations will be submitted by interested ISPCTN sites and will be evaluated by the Data Coordinating Operations Center (DCOC). Selection will occur and ISPCTN sites will be notified.

Provider eligibility: Providers of the eligible practices will be included if:

- they manage ADHD care in patients age 5-11 years
- provide informed consent
- agree to use the RAMP platform if they care for patients randomized to use it
- agree to provide information to potential participants about the study and contact the research coordinator if interested

ADHD Best Practices education at the start of the project will include titration and monitoring guidelines from the current AAP practice guidelines. In the same training session, providers will be educated about accessing the RAMP Reports, oriented to the potential data provided, and instructed on documenting their review in the platform. Providers will be given workflow instructions to obtain the RAMP Reports from teachers. Participating practices will receive compensation of $4000 for their time and engagement working on the study. New providers joining a practice who meet these eligibility criteria will be consented and trained as above at the time of onboarding.

5.2 EXCLUSION CRITERIA

Participants will be excluded if:

- **Child**
  - has serious mental health comorbidities (Children with depression, anxiety, oppositional defiant disorder will be permitted to participate. Children with schizophrenia, bipolar disorder, conduct disorder and those who have been hospitalized for any mental health condition will be excluded from the study.)
  - has severe neurodevelopment disorders (Children with well-controlled seizure disorders will be permitted to participate.)
  - is currently receiving, or previously received, atypical antipsychotic medication treatment
  - is or becomes pregnant
- **Caregiver**
  - none
- **Provider**
  - none

5.3 LIFESTYLE CONSIDERATIONS

Not applicable.

5.4 SCREEN FAILURES

The study team will consider the caregiver/child dyads who undergo screening but who do not enroll in the trial to be screen failures, including those who are contacted by the study team but who do not meet inclusion and/or exclusion criteria. Research coordinators must document and retain the reason for screen failure.

5.5 STRATEGIES FOR RECRUITMENT AND RETENTION

5.5.1 RECRUITMENT OF POTENTIAL PARTICIPANTS

Research coordinators will work with practice providers to schedule the ADHD Best Practice education, RAMP platform orientation, and informed consent encounters for providers who care for children with ADHD at each practice. ADHD Best Practice education and RAMP platform orientation will introduce providers to the study in a group setting. Providers will have an opportunity to ask questions and sign the consent documents at that time or schedule private consent encounter with a research coordinator.

Providers will notify research coordinators of potential participants following an initial evaluation visit for ADHD. Research coordinators may also search for potential participants in the EMR. A partial HIPAA waiver will be obtained for pre-screening procedures used to identify potential participants. Upon return visit and completion of ADHD diagnosis and initiation of stimulant therapy, caregiver/child dyads will receive a flyer from the provider and, if amenable, be referred to a research coordinator for remote review of the project to assess their interest. Research staff will contact interested caregivers by phone, email and/or text. If caregivers are unreachable by any of these methods at first contact, research staff will follow up with at least two additional outreach attempts by one or more methods (phone, email, and/or text) during the first five weeks after stimulant medication is initiated. After a detailed discussion of the project, if a caregiver does not want to participate, the research coordinator will ask reason for refusal and document this information (no PHI required). For all interested caregivers, the study team member will complete the eligibility screening and alert PI to review the eligibility (if needed). Enrollment procedures will include informed consent, HIPAA and FERPA authorizations, orientation to the platform and enrollment text, and obtaining demographics and baseline medical history. Research coordinators will also ask for the caregiver's permission to obtain the initial diagnostic assessment from the diagnosing clinician. Eligible caregiver/child dyads will be randomized to either intervention or control group (1:1).

To keep all PHI protected, keep recruitment practices uniform and have a balanced perspective of the study during the consent process, research coordinators will be trained in the specifics of the study procedures. Research coordinators will be certified in GCP and trained in obtaining informed consent remotely. The data manager will review all forms as well as what data is allowable to retain with research coordinators. Role play of recruitment, enrollment and informed consent process by study PI or their designee before study begins,

followed by planned observation for first few enrollments and then periodically throughout the study. Additional details regarding recruitment training will be stated in the MOP.

The Study PI, site PI, and coordinators will regularly meet to review enrollment status. Practice site calls with the site champion and ideally at least one provider may occur during the enrollment period to keep practice staff informed about the progress of the study. Individual assistance for a site will be provided if a site has not been able to enroll at least 4 caregiver/child dyads within 6 weeks of initiating recruitment/enrollment.

5.5.2 SCREENING AND ENROLLMENT PROCEDURES

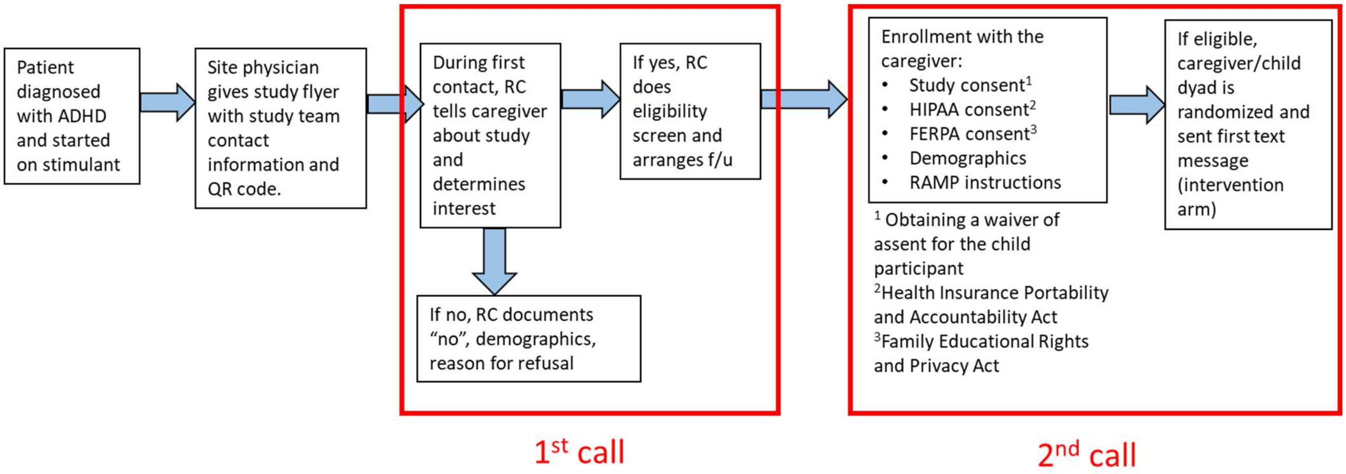

5.5.3 CAREGIVER PARTICIPANT RETENTION

Caregivers and teachers are asked to complete weekly RAMP Reports of the child's ADHD symptoms for 4 weeks followed by monthly RAMP Reports for 2 months. Potential concerns include the length of the RAMP Report and frequency of requests. A text-message-based platform with a direct link to radio-button style responses will be used to decrease this burden. Log-in is not required, and the instrument is formatted to be compatible with smart phone-based usage for completion. Caregiver participants will receive automated reminder prompts at 24 and 48 hours if the RAMP Report has not been completed. Study coordinators will check-in with caregiver participants to ask about any technical difficulties with the RAMP platform or barriers to completing the forms at approximately 1 week, 4 weeks, and 2 months from the time of consent.

Caregivers will be compensated for their time, engagement, and participation in the study. Caregivers will be compensated through ClinCards (or site's preferred method) after completing the enrollment visit ($100), and after completing the 90-day study assessment ($100). Maximum caregiver compensation is $200. ClinCard is similar to a Visa or MasterCard debit card and is used to compensate or reimburse research participants. ClinCard is the preferred method of paying human subjects participating in industry and non-industry clinical trials with recurring visits. ClinCards are provided directly to research participants by the study teams, and the study teams utilize the web-based system to electronically issue payments onto the card. The research

coordinators will dispense ClinCards to participants, track and monitor payments issued, and ensure compliance for the study.

If the 90-day study assessment is not received within 10 days of send-out, then a phone call will be made by the research coordinator with an offer of an additional study assessment provided by the participant's preferred method. This follow-up may be repeated one additional time after an additional 10 days. These study assessments will serve as a marker of retention.

5.5.4 PROVIDER PARTICIPANT RETENTION

Through the study period, the providers will be asked to review the data submitted by caregivers and teachers in the RAMP. An alert will be sent to the provider when new reports have been entered by a caregiver or teacher. The research coordinators will have access to the RAMP provider dashboard in REDCap and will monitor the completion of these review surveys. Each practice site will receive compensation of $4000 for their time and engagement working on the study. Provider participants will receive an automated reminder prompt at 24 hours if the RAMP report review has not been completed. Study coordinators will check-in with provider participants to ask about any technical difficulties with the RAMP platform or barriers to completing the forms at approximately 1 week, 4 weeks, and 2 months from the time of consent. Providers will be compensated for their time, engagement, and participation in the study. Providers will be compensated through ClinCards (or site's preferred method) after signing the provider consent form ($100), and after submitting their last provider survey (attached to the final RAMP report returned for the last child participant assigned to the provider, $100). Maximum provider compensation is $200. Referring potential participants to the study team is a normal activity for providers in clinical trials, and providers who join the study will not receive extra payments for referrals. Providers will not join consent encounters for their patients. Given that providers typically enjoy salaries well above the Unites States median income, the compensation provided should not unduly influence their decision to participate.

6 STUDY INTERVENTION

6.1 STUDY INTERVENTION(S) ADMINISTRATION

6.1.1 STUDY INTERVENTION DESCRIPTION

Research Coordinator Procedures: A research coordinator will be identified at each of the participating ISPCTN sites to organize study procedures at the practices in their state. In the first phase, they will work with site champions at the practices to assist with scheduling the ADHD Best Practice education, RAMP platform orientations, and informed consent encounters for all providers who care for children with ADHD. In the second phase, they will describe the study to interested caregivers and complete enrollment and randomization procedures as well as complete periodic chart reviews and participate in study-related calls. Prior to the start of the first phase, they will receive training in obtaining informed consent if not experienced, given scripted language to use with families when describing the study and supporting caregivers and providers who have not completed or reviewed reports, respectively.

Provider Training: All providers who care for children with ADHD within the study practices will be offered ADHD Best Practice education and RAMP orientation as an introduction to the study. Providers will provide informed consent prior to the start of caregiver/child recruitment and enrollment. This will ensure that there is current knowledge of best practices for ADHD diagnosis and care available to all children receiving care for ADHD within this study. The project is not focused on provider training, as these are not new guidelines, but rather addresses other potential barriers that are unique to rural settings. In addition to this clinical training module, all providers will be required to complete training in the provider use of the RAMP platform.

The RAMP Platform: The technology platform used for this study will be an adaptation of an existing program used for behavioral health screening for pregnant women. This program has screened close to 400 women and has shown superior clinical outcomes compared to usual care.[9,10] The RAMP will be housed in REDCap, a HIPAA-compliant data collection tool. The intervention cohort will be notified of their randomization assignment with a text message or email. They will receive text message requests to fill out the RAMP Reports below. Their responses will be compiled into a RAMP Report which will be available to the provider on their dashboard of enrolled participants.

RAMP Reports completed by caregivers: The RAMP caregiver interface includes monitoring of ADHD symptoms, engagement in behavioral health care visits, and progress in educational settings. These domains of information have been selected as they are typically combined by providers when making decisions regarding medication dose adjustment. The requests for reports will be sent to caregivers as a link in a text message. Upon clicking the link, they will be able to select radio button responses to questions directly on their mobile device (without any login requirement). The RAMP Report requests will be sent weekly for 4 weeks then every month for the remaining 2 months. RAMP Reports will be submittable for 72 hours after initial release. Once a new RAMP Report is available, caregivers cannot go back and complete reports for prior periods.

RAMP Reports completed by teachers: Caregivers in the intervention group will provide teachers with a secure, unique link to access the RAMP Platform. This link will be in the form of a QR code which can be printed on paper or emailed directly to the teacher from the caregiver. Teachers will enter the symptom information directly into the platform. The teacher's RAMP Reports are identical to caregiver RAMP Reports, will consist of the first 18 inattention/hyperactivity questions on the Vanderbilt Assessment, and will be requested at the same interval as the caregivers. We will use an approach similar to that used by Power *et al*.[40] The responses to the teacher RAMP Reports will be available to the providers on the RAMP portal. If a child's teacher does not want to use the RAMP platform, the child will not be disqualified. If a child has multiple teachers, the caregiver should choose one teacher to participate, preferably the one that spends the most time with the child.

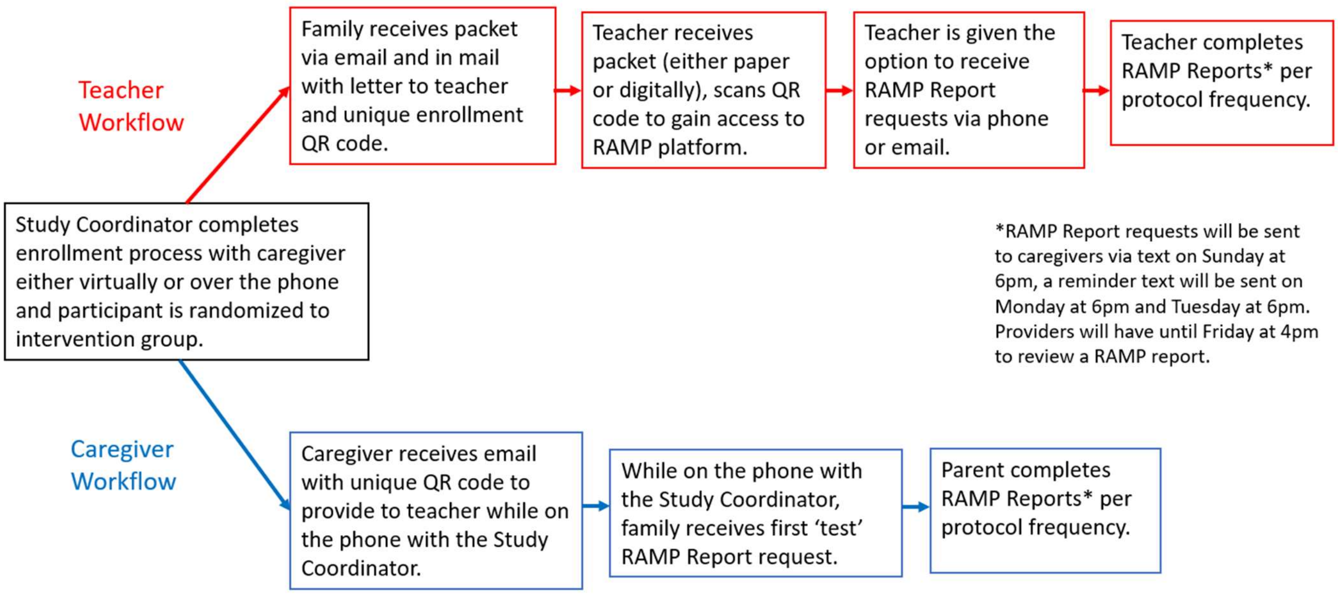

Provider Report Review: Providers will be alerted by their preferred means (text or email) when new caregiver or teacher reports are available for review. While providers will have access to the platform at all times, there will be one provider survey to document review for any given week that there is either a caregiver survey, teacher survey or both to review. .

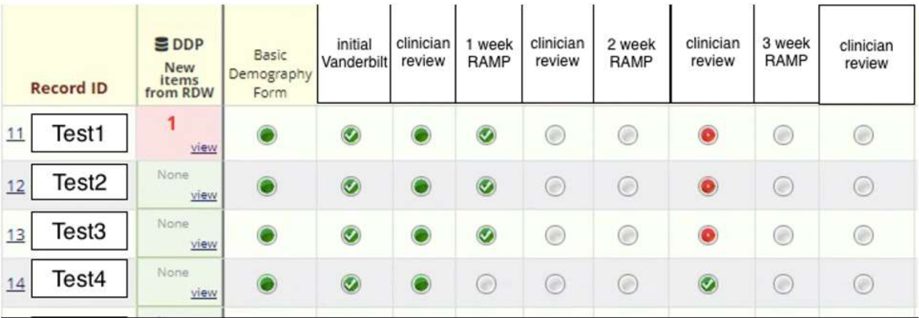

RAMP Platform Prototype. A prototype of the dashboard that providers and research coordinators will use to review participant-entered data and monitor study activities, respectively

The reports submitted by caregivers will be found on a different tab than those submitted by a teacher (but linked with the same ID) to clearly identify source. Providers and research coordinators will review RAMP Reports from a dashboard similar

to the figure on the right. A red dot will indicate an unreviewed RAMP Report has been entered by the participant. The provider will select the red dot to open the report from the caregiver or teacher. Following review, the provider will answer a quick 2-question, radio-button style survey regarding their response to the report. Research coordinators will be able to monitor data entry, and review and document clinical changes as a result of the report.

Digital Education Handouts: Digital education handouts will be sent to all caregivers in the control group on a weekly basis for 4 weeks, then monthly for 2 months. These will be sent through email, and will cover a range of general pediatric topics, such as nutrition, exercise, and screen time.

6.2 MEASURES TO MINIMIZE BIAS: RANDOMIZATION AND BLINDING

Following enrollment, caregiver/child dyads will be randomized into one of two treatment arms:

1. Intervention Arm: will participate in the RAMP for a period of 3 months
2. Control Arm: standard of care determined by the treating healthcare provider; digital education handouts (attention controls) distributed by the study team at the same intervals as the RAMP Report requests in the intervention group

Caregiver/child dyads who complete screening and enrollment procedures will be randomized using a stratified randomization scheme together with a varying permuted block randomization. Enrolled caregiver/child dyads from the practices will be randomly assigned to RAMP (intervention) or standard of care (control) at a 1:1 allocation ratio. Randomization will be stratified by practice (i.e., randomized within practices) to control for potential differences in study population or differences in management. Secure, practice-specific randomization lists will be generated for each site to use when randomizing a participant. At enrollment, the research coordinator will assign randomization assignments in sequential order, pulled directly from the list. Due to the nature of the intervention, it is not possible for blinding of treatment assignments to be performed in this study. All providers will receive Best Practice Education based on the 2019 AAP clinical practice guidelines, including the recommended frequency of for symptom gathering that is the basis for the planned schedule of RAMP Report requests. Therefore, providers caring for children assigned to the control group may attempt to meet this schedule. However, they will still face hardships inherent to paper forms.

Research coordinators will also make note if any paper symptom assessments are returned for the intervention arm in addition to the RAMP reports. A study-specific assessment will be done, at 90 days and will serve as a marker of retention.

6.3 STUDY INTERVENTION COMPLIANCE

The study team will measure and monitor compliance by reviewing the reports submitted by caregivers and teachers and the reports reviewed by providers. The study team will summarize the RAMP utilization as follows:

- Cumulative number of RAMP Reports completed by caregivers
- Cumulative number of RAMP Reports were completed by teachers

- Cumulative number of RAMP Reports were reviewed by providers

The submission of reports will be time-stamped in the REDCap database system. Additionally, caregivers will be asked to provide the diagnostic assessments at study enrollment, and caregivers will be asked to submit a study assessment at the study end-point (at 90 days).

7 STUDY INTERVENTION DISCONTINUATION AND PARTICIPANT DISCONTINUATION/WITHDRAWAL

7.1 DISCONTINUATION OF STUDY INTERVENTION

A participant may be discontinued from study intervention by the site PI. For this study, participants will be discontinued if any of the following occur. Participants may be withdrawn from study due to an AE/SAE, or if remaining in the study is no longer in their best interest. Given the age of participants in this study, pregnancy is unlikely. However, in the unlikely event pregnancy were to occur, the child would be withdrawn from the study. While the RAMP intervention would not affect a pregnancy in any way, a patient would likely be referred to a psychiatrist or obstetrician for stimulant cessation or management.

7.2 PARTICIPANT DISCONTINUATION/WITHDRAWAL FROM THE STUDY

Participants (caregiver/child dyads) may choose to withdraw from the study at any time. Their end date and reason for withdrawal if shared will be noted in OpenClinica. Those who exit the study will return to usual, provider-directed care. Their access to the platform will be discontinued.

The study team will consider the following to be an early-terminated participant: any caregiver who completes the informed consent process and enrolls in the study but later withdraws their consent, or the site investigator removes the participant from the study. Participants may withdraw from the study at their own request (e.g., participant choses to decline any further ADHD treatment), or the site investigator, Data Safety Monitoring Board (DSMB), or DCOC can withdraw a participant. If a participant withdraws from the study, site coordinators or investigators can ask the participant why they chose to withdraw. Research coordinators will document the reason for withdrawal.

The text messages sent from the RAMP will give recipients the option to reply "STOP" to opt-out of the text messaging service. If a caregiver or provider participant opts-out of the text messaging service in this way, a study coordinator will reach out to them to confirm they no longer want to receive text messages from the platform. Participants that confirm they wish to opt-out of receiving text messages will be withdrawn from the study. Withdrawing a participant from the study will not remove the information already collected about that participant from the study. Teachers will not be contacted if they opt-out of receiving text messages from the RAMP, and this will not affect the caregiver/child dyad's participation in the study.

If a provider participant leaves the practice or withdraws consent to participate, a phone call with the principal investigator, provider, and research coordinator will be held. All children enrolled in the study who are cared for by the provider will be reviewed to determine strategies for continuation in the study.

7.3 LOST TO FOLLOW-UP

The study team will consider caregiver/child dyad participants lost to follow-up if they do not complete the final study assessment. Those who do not complete any clinical assessments (RAMP Reports or paper assessments)

will still be included if they complete the final study assessment as this is an intention to treat analysis. To minimize the number of participants lost to follow-up, the study team will contact participants approximately 1 week, 4 weeks, and 2 months after consent to ensure there are no technology issues.

8 STUDY ASSESSMENTS AND PROCEDURES

8.1 EFFICACY ASSESSMENTS

PRIMARY OUTCOME MEASURES

Objective 1 – Evaluate completion of clinical assessments by caregivers and teachers.

The number of completed clinical assessments (either paper assessments [control] or RAMP Reports [intervention] that are returned to providers during the 3-month study period will be counted. Each RAMP Report will be documented in the RAMP platform including a timestamp of submission. Throughout the study period, the research coordinators will facilitate recruitment, randomization, support retention, and perform chart abstraction.

SECONDARY OUTCOME MEASURES

Objective 2 – Evaluate provider acceptability of the RAMP through utilization measures.

Each report that the provider completes to document review of a report will be available in the RAMP platform along with a timestamp of submission. The research coordinators will verify that requests have been distributed through the RAMP platform. Only data reviewed by providers without research coordinator prompting will count towards the proportion of reports reviewed. If outreach is provided by research coordinator or Study PI, this will occur outside of the time window outlined in the endpoints and will be for the purposes of ensuring good patient care. The secondary endpoint, proportion of provider review surveys with at least one available RAMP Report where review is documented, is only relevant to the subset of participants randomized to the RAMP intervention group. There is no formal hypothesis testing for this secondary objective.

8.2 SAFETY AND OTHER ASSESSMENTS

Because this study is of an educational intervention, adverse events (AEs) and serious adverse events (SAEs) are not expected. While the study team will not solicit AEs or SAEs, the study team will provide all participants with a telephone number to report AEs/SAEs. AEs and SAEs will be collected for all participants.

8.3 ADVERSE EVENTS AND SERIOUS ADVERSE EVENTS

8.3.1 DEFINITION OF ADVERSE EVENTS (AE)

An AE is defined as any untoward occurrence associated with the use of an intervention in humans, whether or not considered intervention related (21 CFR 312.32 [a]). Stable chronic conditions that present prior to enrollment and do not worsen are not considered AEs but will be accounted for in the participant's medical history. Exacerbation or worsening of pre-existing conditions are defined as AEs. Reported AEs will be reviewed and classified by the investigator. We will only record and track severe, serious, and AEs related to the study

intervention. Expected AEs are listed below (8.3.3.3). This study is only reporting AEs for the child participant due to the negligible risk to providers and caregiver participants.

8.3.2 DEFINITION OF SERIOUS ADVERSE EVENTS (SAE)

We will consider an AE or suspected adverse reaction "serious" if, in the view of the PIs, medical monitor, DSMB, or sponsor, it results in any of the following outcomes:

- Death
- Life-threatening AE
- Inpatient hospitalization or prolongation of existing hospitalization
- Persistent or significant incapacitation or substantial disruption of the ability to conduct normal life functions
- Congenital anomaly/birth defect.

Important medical events that may not result in death, be life-threatening, or require hospitalization may be considered serious when, based upon appropriate medical judgment, they may jeopardize the participant and may require medical or surgical intervention to prevent one of the outcomes listed in the SAE definition.

8.3.3 CLASSIFICATION OF AN ADVERSE EVENT

The investigator or qualified sub-investigator is responsible for assessing all AEs for causality and severity, and for final review and confirmation of accuracy of event information and assessments.

8.3.3.1 SEVERITY OF EVENT

These are the guidelines to describe the severity of AEs:

Mild. Events require minimal or no treatment and do not interfere with the participant's daily activities.

Moderate. Events result in a low level of inconvenience or concern with the therapeutic measures. Moderate events may cause some interference with daily functioning.

Severe. Events interrupt a participant's usual daily activity and may require systemic drug therapy or other treatment. Severe events are usually potentially life-threatening or incapacitating. Of note, the term "severe" does not necessarily equate to "serious."

8.3.3.2 RELATIONSHIP TO STUDY INTERVENTION

All AEs and SAEs must have their relationship to trial intervention assessed by the clinician who examines and evaluates the participant based on temporal relationship and her/his clinical judgment. We will grade the degree of certainty about causality by using the categories below.

Related. We know the AE occurred with the trial intervention, there is a reasonable possibility that the trial intervention caused the AE, or there is a temporal relationship between the trial intervention and event.

Reasonable possibility means that there is evidence to suggest a causal relationship between the trial intervention and the AE.

Not Related. There is not a reasonable possibility that the administration of the trial intervention caused the event, there is no temporal relationship between the trial intervention and event onset, or there is an established alternate etiology.

8.3.3.3 EXPECTEDNESS

The site investigators will be responsible for determining whether an AE or SAE is expected or unexpected.

An AE or SAE will be considered unexpected if the nature, severity, or frequency of the event is not consistent with the risk information previously described for the trial intervention.

Expected events could include the following: medication related side effects (sleep difficulty, appetite suppression, stomachache, headache) as well as new mental health diagnoses (depression, anxiety, suicide attempt, substance use disorder).

8.3.4 TIME PERIOD AND FREQUENCY FOR EVENT ASSESSMENT AND FOLLOW-UP

The study team will collect severe AEs, SAEs, and AEs related to study procedures. The study team will provide participants with a site-specific telephone number to report AEs, but will not actively solicit AEs or SAEs. The occurrence of a SAE may come to the attention of study personnel during clinical visits, by the clinical team with administration of RAMP Reports, or by the medical monitor upon reviewing data. The site study team will capture all AEs on the appropriate case report form following the instructions and timelines provided in the MOP. Information to be collected includes event description, date/time of onset, date/time of resolution, clinician's assessment of severity, relationship to study intervention and time of resolution/stabilization of the event. Site study teams must follow all severe and SAEs, regardless of relationship, until there is resolution, the condition stabilizes, the event is otherwise explained or is judged by the protocol study team to be no longer clinically significant, or the participant is lost to follow-up.

Clinic providers participating in this study will be asked to remind caregiver participants to report any medication side effects or events that caused them to seek medical assistance or hospitalization for their child to the study team.

8.3.5 ADVERSE EVENT REPORTING

Site coordinators and/or the study team will record potentially related AEs and severe AEs in the electronic data capture (EDC) system, include them in the statistical analysis, and follow the reporting procedures outlined in the trial-specific MOP. The MOP section on reporting severe AEs will encompass the requirements of:

1. Reviewing IRB's policies and procedures
2. Standard Operating Procedures (SOPs) for the Streamlined, Multisite, Accelerated Resources for Trials IRB Reliance platform (SMART IRB)

3. ICH E6(R2), GCP: Integrated Addendum to ICH E6(R1): Guidance for Industry
4. Following local IRB's policies and procedures, when applicable. If there are discrepancies between the procedures, we will follow the most stringent of the procedures.

The DCOC will report severe AEs to the reviewing IRB for its yearly continuing review. The DCOC will report to the Central IRB (cIRB), as specified in the study-specific IRB communication plan, based on recommendations of the SMART IRB. Sites will also report severe AEs to the local IRB, per local IRB policies and procedures, and make them available to the sponsor (DCOC) on a continuing basis through the EDC system. The study team will report to the DSMB, per the DSMB charter.

The trial-specific MOP will describe the details of the reporting structure and additional details related to timelines for reporting.

The specific regulations of the FDA do not apply to this protocol because this is not an FDA-regulated trial.

8.3.6 SERIOUS ADVERSE EVENT REPORTING

Site coordinators will record all SAEs in the EDC, and the DCOC will report SAEs, per Table 4 below. The DCOC will notify monitoring personnel (e.g., medical monitor) and monitoring bodies (e.g., DSMB), per the DSMB charter.

Table – Serious Adverse Event Reporting.

Report to	Timing	Notes
Reviewing IRB (SMART IRB definition)	Yearly (minimum)/at continuing review	For continuing review; Submitted to IRB via DCOC
Local IRB	Per local IRB policies and procedures	
DCOC (Sponsor, per SMART IRB definitions, and lead trial team)	Immediately, but no later than 48 hours after finding out about the event	CO-SOP-012.v1.0/SOP
Overall PIs (per SMART IRB definitions)	Immediately, but no later than 24 hours after finding out about the event	
Medical Monitor	Per DSMB charter	
DSMB	Per DSMB charter	
NIH Project Scientist	Per DSMB charter	

All SAEs will be followed until satisfactorily resolved, the site investigator deems the event is chronic, the participant is stable, or the participant is lost to follow-up. The DCOC/trial sponsor may request other supporting documentation, and sites should provide this as soon as possible.

The DCOC will ensure the summary and report of SAE data to the reviewing IRB in time for consideration at the next continuing review. The DCOC will also create and provide any SAE data summaries requested by the

monitors or specified in the DSMB charter. The site investigators will be responsible for following their local institution's requirements.

The trial-specific MOP has additional details.

8.3.7 REPORTING EVENTS TO PARTICIPANTS

The study team will notify participants of those trial-related (or potentially trial-related) SAEs that may affect their willingness to continue with the trial or the future health of the participant. Any of the following can determine if study personnel should contact participants: the IRB, the medical monitor, the DSMB, or the PIs. The person or oversight body that makes the determination will inform the DCOC, which will instruct the site investigators and site coordinators to contact the participants consented through their site.

Site investigators and/or site coordinators will record any contact with participants, if necessary, in the EDC and/or trial log.

8.3.8 EVENTS OF SPECIAL INTEREST

We have not identified other reportable events for this trial.

8.3.9 REPORTING OF PREGNANCY

Given the age of the child participants in this study, pregnancy is unlikely. In the unlikely event pregnancy were to occur, the child would be withdrawn from the study. Child participants' pregnancies will be reported in the EDC but not followed for the pregnancy duration. While the RAMP intervention would not affect a pregnancy in any way, a patient would likely be referred to a psychiatrist or obstetrician for stimulant cessation or management.

8.4 UNANTICIPATED PROBLEMS

8.4.1 DEFINITION OF UNANTICIPATED PROBLEMS (UP)

The Office for Human Research Protections (OHRP) considers unanticipated problems involving risks to subjects or others (UPIRTSOs) to be any problem, event, or new information that is:

- Unanticipated or unexpected;
- Related to the research, and
- Involves new or increased risks to subjects or others.

A UPIRTSO is not necessarily an AE or an SAE. For example, a breach of confidentiality is a potential UPIRTSO that is not an AE or SAE.

The trial-specific MOP provides additional details.

8.4.2 UNANTICIPATED PROBLEM REPORTING

Site investigators and/or site coordinators must report potential UPIRTSOs, per this protocol, to the reviewing IRB, via the method(s) specified in the MOP, and directly to the DCOC. If the UAMS IRB determines the issue is, indeed, a UPIRTSO, the DCOC will notify persons and entities as required by UAMS IRB policies and procedures, the study-specific communication plan, and the DSMB charter. Site investigators and/or site coordinators must also report these events to their local institutions according to the rules and regulations of the local institution. The reviewing IRB will report the issue to OHRP, per the policies of the reviewing IRB. For the UAMS IRB, which will be the reviewing IRB for sites in this trial, the required reporting times are provided in the contemporaneous version of 10.2, *Information that must be reported to the IRB and IRB actions*, which is available via http://irb.uams.edu/irb-policies/current-irb-policies/principal-investigator-responsibilities/. The reporting times, at the time of the approval of this protocol, are in the table below.

Table – Unanticipated Problem Reporting (per UAMS IRB policy at time of approval of this protocol).

Unanticipated Problem	Required Reporting Time to UAMS IRB
Death or life-threatening	Immediately to IRB office or IRB Chair
All other events	Within 10 days of event or notification of event if non-local

8.4.3 REPORTING UNANTICIPATED PROBLEMS TO PARTICIPANTS

The participants will be notified of those study-related (or potentially study-related) SAEs and unanticipated problems (UPs) that may affect the participants' willingness to continue with the study or the future health of the child enrolled in the study. This determination can be made by any of the following: the IRB, the medical monitor, the DSMB, or the sponsor. The person or oversight body that makes the determination will inform the DCOC, which will instruct the site PIs/research coordinators to contact those participants enrolled through their site. Contacts with participants, if necessary, will be recorded on the appropriate CRF and/or study log.

9 STATISTICAL CONSIDERATIONS

All statistical analyses will be detailed in a separate Statistical Analysis Plan (SAP). This plan may be revised during study implementation to accommodate protocol amendments and to make necessary changes to unexpected issues in study execution and data that impact planned analyses. A finalized SAP will be issued prior to data lock.

9.1 STATISTICAL HYPOTHESES

Primary Endpoint:

H_1: Use of the RAMP platform will result in significantly greater number of completed clinical assessments per participant in the intervention group compared to the control group

9.2 SAMPLE SIZE DETERMINATION

A total sample of 36 participants was agreed in accordance with published guidance for pilot studies. Up to 36 caregiver/child dyads and 20 providers will be enrolled. Our proposed sample size is also consistent with the recommendations of Whitehead and colleagues (2016).[41] Their derived new stepped rules of thumb for a pilot trial sample size vary depending on an estimated standardized effect size for the main efficacy trial; therefore, if we assume a medium Cohen's effect (i.e., $0.3 \leq \delta \leq 0.7$) and 90% power for the main trial, a sample size of 36 participants with 18 randomized to each intervention arm in the pilot study is sufficient.

With a focus on participant recruitment and adherence, a sample size of 36 allows the study team to estimate an expected recruitment rate of 25% and adherence rate of 50%, each within 12-14 percentage points, with a confidence level of 90%.[42] We expect the rate of return of the RAMP Reports to be a comparable rate to the general survey response rate of 52.7%, though it may be higher as the platform aims to function as an engagement tool.[43]

9.3 POPULATIONS FOR ANALYSES

To evaluate completion of clinical assessments and provider acceptability, the study will have two analysis populations:

1. Intention-to-treat (ITT) population – This population will include all participants randomized into the study.
2. Per-protocol (PP) population – This population will include all participants in the ITT population who completed the final study assessment.

9.4 STATISTICAL ANALYSES

9.4.1 GENERAL APPROACH

Following finalization of the protocol, but prior to data lock, the DCOC statistical team will issue a Statistical Analysis Plan (SAP) as a separate document, which will provide detailed analytical plans for the set of analyses outlined below. The statistical team will conduct all statistical analyses following the statistical principles for clinical trials as specified in ICH Statistical Principles for Clinical Trials (ICH Topic E9). The study team will describe and justify any deviations from the planned analyses in the final integrated clinical study report. The study team will present all study data and summary tables for the overall study and by study sites.

Descriptive data summaries for continuous data will include mean and standard deviation or median and interquartile range, as appropriate. The team will also summarize categorical data by using frequency and proportion, and they will investigate any outliers detected during data review and will define in the SAP methods for handling outliers or data transformation.

9.4.2 ANALYSIS OF THE PRIMARY ENDPOINT(S)

Determining the utilization of clinical assessments (paper assessments in control group and RAMP Reports in intervention group) in rural primary care clinics will be a key parameter necessary for conducting a future main trial. Utilization will be determined by assessing the numbers of completed caregiver reports and teacher reports in each of the groups (intervention and control). Counted reports include any clinical assessment including paper assessment or RAMP Report that is submitted to a provider but not the midpoint and endpoint study assessment that will be for study purposes only. Any paper assessments submitted for intervention group participants will be counted separately from the RAMP reports and a sensitivity analysis will be performed to gain insight into that data. Data analysis will report mean, standard deviation, median, and interquartile range for each utilization measure overall and stratified by intervention group. Additionally, we will develop a Poisson regression model to compare the number of returned structured reports across the groups. We will report the expected mean number of reports in each group along with their difference with 95% confidence interval (CI).

9.4.3 ANALYSIS OF THE SECONDARY ENDPOINT(S)

The secondary endpoint, proportion of provider review surveys with at least one available RAMP Report where review is documented, is only relevant to the subset of participants randomized to the RAMP intervention group. There is no formal hypothesis testing for this secondary objective. The proportion of provider review surveys with at least one available RAMP Report where review is documented will be estimated along with the corresponding 95% confidence interval using the Wilson score interval or other method appropriate to the data characteristics.

9.4.4 SAFETY ANALYSES

The DCOC will detail reported intervention-related AEs and SAEs experienced by study participants and will summarize these by the intervention arm. The DCOC will present summary statistics for overall and by sites.

9.4.5 BASELINE DESCRIPTIVE STATISTICS

The statistical team will summarize baseline and demographic characteristics by intervention group and will apply summary statistics for both continuous and categorical variables. For continuous variables, descriptive measures will include number of non-missing values, mean, standard deviation, median, minimum, and maximum. For categorical variables, descriptive statistics will include frequency counts and proportions by category.

9.4.6 PLANNED INTERIM ANALYSES

There are no planned interim analyses for this study.

9.4.7 SUB-GROUP ANALYSES

No sub-group analyses will be performed.

9.4.8 TABULATION OF INDIVIDUAL PARTICIPANT DATA

The statistical team will provide a detailed description of participant disposition, will tabulate the number of enrolled participants overall and by study site, and will present this data as counts and proportions. Additionally, the statistical team will summarize the number of participants either completing or discontinuing the study using counts and proportions.

9.4.9 EXPLORATORY ANALYSES

We will use a Wilcoxon rank-sum test to compare the number of caregiver specific returned structured reports in each group. Additionally, we will repeat the same analysis for the number of teacher-specific returned structured reports in each group.

10 SUPPORTING DOCUMENTATION AND OPERATIONAL CONSIDERATIONS

10.1 REGULATORY, ETHICAL, AND STUDY OVERSIGHT CONSIDERATIONS

10.1.1 INFORMED CONSENT PROCESS

Informed consent is a process that starts before a participant agrees to participate in the trial and continues throughout the individual's trial participation. For sites using the UAMS IRB as their reviewing IRB, the contemporaneous version of UAMS IRB policy 15.5, Informed Consent Process, is applicable and must be followed. The policy is available at https://irb.uams.edu/irb-policies/current-irb-policies/. If there are discrepancies between this protocol and applicable reviewing IRB policy, the more stringent requirement will apply.

Participants. Participants will include both the child and their caregiver as a dyad as well as all providers managing ADHD at a participating clinic. The participating caregiver is defined as the child's parent/LAR. ISPCTN coordinators or research-trained designees will obtain provider consent prior to the start of caregiver/child dyad enrollment.

Full waiver of the assent process: We are requesting a full waiver of assent for the RAMP study based on the following justifications:

- The child has no active involvement and the study will not change the child's normal activities. We will only be collecting their medical information.
- Medication changes that may evolve from symptom information delivered electronically are entirely at the physician's discretion, and will not be influenced by the study team.
- While it's preferable for a child to be present during the consent discussion, the study's consent process may occur in person or remotely, and it could be harder to keep a child's attention over the phone/remotely. This might cause an imbalance in how well children absorb the information between the two arms because it's a small study (36 dyads).

If the full waiver of assent is granted, child participants will not be asked to provide assent in order to participate in this study.

Engaging with the School Environment: It is a guideline recommended practice for ADHD symptom information to be requested from teachers in addition to caregivers. Teachers of children receiving usual care will obtain/complete/return paper symptom assessments in the usual way (as directed by the caregiver). The study team will then use the clinic's medical record to document paper assessments returned by teachers for usual care. Teachers of children assigned to the RAMP intervention will receive instructions from caregivers on how to create a RAMP user account and complete RAMP Reports. The study team will provide caregivers with information packets to give to teachers assigned to the RAMP arm (one teacher per child). Information packets will include a brief description of the purpose of the study, FERPA authorization (to share information directly

with the study, see section 10.1.1.1 and 34 CFR 99.30), and a QR code with instructions to connect teachers with the RAMP platform. RAMP Report requests will be delivered to teachers by automated text message, and their reports will be completed directly in the RAMP system. The study team will use the RAMP system to document RAMP Reports returned by teachers. The ADHD symptom questions teachers will answer in the RAMP Report are consistent with standard practice, are identical to the caregiver RAMP Report, and will be used by participating providers for the child's ADHD care management. Teachers must provide a phone number capable of receiving text messages and an email address.

Teachers will not complete study assessments, and their impressions of child behavior contained in the paper assessments or RAMP Reports will not be recorded by the study team outside the medical record or RAMP platform. For these reasons, teachers will not be considered research participants. No direct contact is planned between the study team and teachers. Teachers will be directed to contact caregivers if they need assistance with the study. If a child's teacher does not want to use the RAMP platform, the child will not be disqualified from the study.

10.1.1.1 CONSENT AND OTHER INFORMATIONAL DOCUMENTS PROVIDED TO PARTICIPANTS

All participant-facing documents will be approved by the IRB prior to distribution to participants. These documents include informational flyers, scripted communications, educational/instructional materials, informed consent forms, HIPAA form, and research FERPA form. Consent process forms will describe the study intervention, each participant's role/procedures in the study, as well as the foreseeable risks and benefits, and available compensation, where applicable.

FERPA authorization. Caregivers may be asked to provide a signed clinical FERPA form to teachers in the control arm, authorizing the teacher and provider to share information. This practice varies between clinics, so the study team will not request that clinics change their usual practice/documentation regarding FERPA authorization. The study will not seek IRB approval for clinical forms used for usual care (clinical FERPA and paper symptom assessments). Teachers who are asked to submit symptom assessments directly in the RAMP system will receive a research FERPA form (signed by the caregiver) that will authorize the teacher and study team to share information, and will describe the purpose of the study. The original research FERPA form will be kept by the study team and copies will be given to the caregiver and teacher/school. Teachers in the RAMP arm will also receive a QR code to allow them to access the RAMP and receive RAMP Report requests via text message.

10.1.1.2 CONSENT PROCEDURES AND DOCUMENTATION

The participating caregiver is defined as the child's parent or legal guardian/LAR. The study team will confirm that any caregiver participant has the current legal authority to consent and provide parental permission for the child.

Recruitment and confirmation of eligibility will proceed as described in sections 5.5.1 and 5.5.2. Once deemed interested and eligible, the study team member will conduct the informed consent process with the participant.

The study team will collect written informed consent, HIPAA authorization, and FERPA authorization from caregivers. Sites will return a signed copy of all consent, FERPA (research) and HIPAA authorization forms given to the participants. Study teams must document the informed consent process, following the current UAMS Institutional Review Board policy.

Site research staff will ask participants to read and review the consent forms and HIPAA authorization document(s). Site research staff will explain the trial to the participant(s) in terms the participant(s) can understand and answer any questions that may arise. The explanation will state the purposes, procedures, and potential risks of the trial and describe participants' rights as research participants. Research coordinators will inform all participants that participation is voluntary, that they may withdraw from the trial at any time without prejudice, and that nonparticipation will not adversely affect their medical care or employment with their clinic. Participants will have the opportunity to carefully review the consent form and ask questions before signing. The site research staff will give participants the opportunity to discuss the trial with their family or surrogates or think about participating in the trial before agreeing to participate. The participant must sign the informed consent form prior to any study-specific procedures being done. When sites receive a participant's signed consent form, the study team member who participated in the consent discussion will countersign the consent form and return a copy of the countersigned form to participants. Participants will be asked to keep a copy of the consent form that is signed by all parties. Once the site and participants complete this process, the study team will consider the participant enrolled in the study.

The study team member who participated in the consent discussion will document the consent processes in the source documents. Consent documentation will include, at a minimum:

1. the title of the trial,
2. the date the participant entered into the trial,
3. the name of the site investigator
4. the name of the person(s) obtaining the informed consent, and
5. a statement that the caregiver of the participant received a copy of the signed form.

The following additional documentation is recommended, but it is not required:

1. a list of who else was present during the process,
2. the type of questions asked by the caregiver of the participant,
3. a summary of details that demonstrate the caregiver of the participant understood the information, and
4. a description other specific details related to that case.

The informed consent process may be conducted in person or remotely via HIPAA-compliant means (e.g., via US postal service, telephone, REDCap, or video conference platform, etc.). Consenting procedures will conform to the site's and UAMS central IRB's approved procedures, and the more stringent requirement will apply. The remote consent process will parallel the consent process used for in-person consenting. The only difference will

be the method(s) of communication. The participant must sign the informed consent form prior to any study-specific procedures being done. The study team will ensure that, as with in-person consenting, the participant is given sufficient opportunity to ask questions, is able to understand the nature of this study and what participation entails, and is provided a copy of the final, completed consent signed by all parties involved, including the research team member who obtained consent and, when applicable, the site investigator. This final, signed consent will be provided via a HIPAA-compliant method or a method that the participant has agreed to in writing. The site research team members working on the consenting process will ensure that any caregiver who is consenting remotely has the authority to consent for the child.

10.1.2 STUDY DISCONTINUATION AND CLOSURE

This trial may also be suspended or stopped per any stopping/suspension specifications in the DSMB charter. Early termination may be permanent if there is sufficient cause.

The suspending or terminating party will provide, directly or indirectly, written notification documenting the reason for trial suspension or termination to the following, as applicable: trial participants, PIs, ISPCTN investigators, reviewing IRB, local IRBs, DCOC, and OHRP. Persons and offices notified will include those specified, the reviewing IRB's policies and procedures, and the SMART IRB policies and procedures.

The suspending or terminating party will also contact trial participants and inform them of any changes to the trial visit schedule. Circumstances that may warrant termination or suspension include, but are not limited to:

- Determination of unexpected, significant, or unacceptable risk to participants
- Demonstration of efficacy that would warrant stopping
- Insufficient compliance to protocol requirements
- Data that are not sufficiently complete and/or evaluable
- Determination that the primary endpoint has been met
- Determination of futility

If the trial is temporarily suspended, it may resume once concerns about safety, protocol compliance, and data quality are addressed and are satisfactory to the DSMB, the reviewing IRB, the local IRBs (when applicable), the funding agency, and the sponsor (DCOC).

10.1.3 CONFIDENTIALITY AND PRIVACY

The study team will record data from the software to a secure HIPAA-compliant database. Sites and study team members will conduct all trial activities in as private a manner as possible.

Records will be maintained as required by the privacy and security rules promulgated by the Health Insurance Portability and Accountability Act (HIPAA) (Title 45 of the CFR Part 164). 55,56

During the trial, site investigators and/or site coordinators will keep all trial records in secure locations that only authorized personnel can access. Examples of secure locations include but are not limited to: 1) locked file cabinet(s) in a limited (badge or key) access room, or 2) password-protected computer systems. Study personnel may only transmit records that contain PHI, as defined by HIPAA, through an open email system if the personnel encrypt the data. Password protection alone is insufficient for data transmission through an open email system (e.g., Outlook). After trial completion, access to trial records will be limited (see next section).

Certain bodies/institutions may need to review information, including the participant information, for any of the following reasons: to process information or to ensure compliance with the protocol and other applicable requirements (such as the policies and procedures of the cIRB). Institutions/bodies that may have access to the participants' information include:

- UAMS IRB (cIRB) and other oversite offices
- IRB for the site through which the participant is consented
- OHRP
- DCOC
- NIH

Individuals with access to trial records will be:

- PIs
- Site PIs
- Site coordinators
- Data managers at participating site(s)

10.1.4 MULTI-SITE COMMUNICATIONS (IRB-RELATED)

This study will be conducted at two sites within the ISCPTN network. All sites will cede to the UAMS IRB as the reviewing IRB (per SMART IRB definitions). The study-specific IRB-related communications plan was constructed from the SMART IRB template and uses SMART IRB recommendations for communications. This plan will be submitted to the IRB as a separate study-specific document. The DCOC will serve as the lead study team and will be the intermediary between the sites and the UAMS IRB as the central (or single) IRB (i.e., cIRB). Other types of communications (i.e., related to data, study deviations, etc.) between DCOC and the sites are detailed in their respective appropriate sections of this protocol.

10.1.5 FUTURE USE OF STORED SPECIMENS AND DATA

No specimens will be collected or stored for this trial. Regarding stored data, site investigators, site coordinators, and study team members will document all trial interactions, and these will be password protected in secured facility/location.

The study team will place participant's de-identified data and other limited information, such as race and ethnic group, into one or more centralized database(s). The study team will share this data in compliance with the NIH data sharing policy.

For future studies using any procedures or analysis not specified in this protocol, IRB approval is required. If another investigator/collaborator has a meaningful purpose for accessing the data retrieved in this protocol, the DCOC must consult the PI and the cIRB must approve.

During the informed consent discussion, caregiver participants will be given the option to consent to be contacted for future research related to this study. If they agree, their information will be securely stored at the participating sites to be used for future contact.

10.1.6 KEY ROLES AND STUDY GOVERNANCE

**Principal Investigator**	
Name	Claire MacGeorge, M.D.
Institution	*Medical University of South Carolina*
Email	*macgeorg@musc.edu*
**DCOC (Overall, cIRB) PI**	
Name	Songthip Ounpraseuth, Ph.D.
Institution	*University of Arkansas for Medical Sciences*
Email	*STOunpraseuth@uams.edu*
**DCOC Co-PI**	
Name	Fred Prior, Ph.D.
Institution	*University of Arkansas for Medical Sciences*
Email	*FWPrior@uams.edu*
**DCOC Co-PI**	
Name	Sherry Courtney, M.D
Institution	*University of Arkansas for Medical Sciences*
Email	*SECourtney@uams.edu*
**Statistical Support**	
Name	Kristin Gaffney
Institution	*University of Arkansas for Medical Sciences*

Email	*KKGaffney@uams.edu*
**Statistical Support**	
Name	Milan Bimali, Ph.D.
Institution	*University of Arkansas for Medical Sciences*
Email	*MBimali@uams.edu*
**Medical Monitor**	
Name	Rebecca Latch, M.D.
Institution	*University of Arkansas for Medical Sciences*
Email	*LatchRebeccaL@uams.edu*

10.1.7 SAFETY OVERSIGHT

Ensuring participant safety is the responsibility of all study team members, especially the PIs, site investigators, site coordinators, and monitors. A medical monitor and the DSMB will provide oversight.

The medical monitor will be a pediatrician with relevant expertise and will be independent of the trial. The NIH will convene the DSMB, and it will meet regularly, according to its charter.

The entities that will receive reports include, but are not limited to, the DCOC and the NIH. The DSMB charter will provide additional information.

10.1.8 CLINICAL MONITORING

Clinical site monitoring will be conducted to ensure that the rights and well-being of trial participants are protected, that the reported trial data are accurate, complete, and verifiable, and that the conduct of the trial complies with the current approved protocol and other IRB-approved documents, with International Council for Harmonisation Good Clinical Practice (ICH GCP E6 (R2)), with applicable regulatory requirements, and other requirements/guidelines necessary to complete study conduct, which includes the trial-specific Manual of Procedures (MOP).

Monitoring for this trial will be performed by DCOC staff or their designee.

Monitoring will be planned and conducted either onsite, remotely, or centrally according to the Site Monitoring Plan for this trial. Monitors will use the Site Monitoring Plan to guide their review and guide the documentation and reporting of their activities and findings. The Site Monitoring Plan describes who will conduct each instance of monitoring, the frequency at which they will be performed, the level of detail that will be applied during review sessions, and details about the distribution of monitoring reports.

10.1.9 QUALITY ASSURANCE AND QUALITY CONTROL

Each IRB-approved site entering data will perform internal quality management of study conduct, data collection, documentation, and completion. Sites that ceded to the UAMS IRB (as central IRB) will follow applicable UAMS IRB policies, available at https://research.uams.edu/irb/policies/current-policies/. A listing of applicable policies will be provided in the MOP. Each site will follow the trial-specific MOP and any additional site-specific SOPs. Each participating site will share site-specific SOPs with the DCOC. The operating procedures will include, but are not limited to, procedures for: 1) performing the consent process; 2) data collection, entry, review, and submission processes; 3) assigning roles and responsibilities of site personnel; and 4) training methods for study staff. Each site will provide direct access to all their facilities, source data/documents, and reports for the purpose of monitoring and auditing by the DCOC and inspection by local and other applicable regulatory authorities. When electronic health records are source data/documents, sites must provide read-only access for the monitors and auditors and anyone else authorized to inspect or verify records.

The DCOC will implement quality control procedures for the database and DCOC-maintained records in accordance with the Site Monitoring Plan, MOP, data safety monitoring plan (DSMP), and applicable SOPs. The DCOC will communicate information about any data anomalies to the site(s) for clarification/resolution.

The DCOC will address issues uncovered during quality assurance, quality control, or monitoring activities through simple corrections or root-cause analysis, followed by corrective and preventative action plans (CAPA), as appropriate according to DCOC SOPs.

10.1.10 DATA HANDLING AND RECORD KEEPING

10.1.10.1 DATA COLLECTION AND MANAGEMENT RESPONSIBILITIES

A formal data management plan will describe and document the data and workflow for the trial. The data management plan and associated documentation will specify all operations performed on data from origination to database lock, including detailed descriptions of source documentation, case report forms (CRF), instructions for completing forms, data handling and record keeping procedures, procedures for data monitoring, and reconciliation procedures and coding dictionaries to be used, if applicable. The data management plan will also describe the specific data collection and management responsibilities required of the sponsor, PIs, site investigators, site awardees, clinics, and DCOC. The data management plan contents will be consistent with those described in the Good Clinical Data Management Practices (GCDMP). The DCOC will provide the data management plan components that document operations performed on the data to the PIs for review and approval prior to implementation.

Data collection is the responsibility of the trial staff at the individual sites under the supervision of the site investigator. The site investigator is responsible for ensuring the accuracy, completeness, legibility, and timeliness of the data reported. All source documents must be completed using standard good documentation practices (i.e., the ALCOA-C method [attributable, legible, contemporaneous, original, accurate, and complete]).

It is best practice for site coordinators to use hardcopies of any data recorded on paper case-report forms or trial visit worksheets/assessment forms as source document worksheets for recording data for each participant consented. Data recorded in EDC derived from source documents must be consistent with the data recorded on the source documents.

To keep all PHI protected, keep recruitment practices uniform and have a balanced perspective of the study during the consent process, research coordinators will be trained in the specifics of the study procedures. Research coordinators will be certified in GCP and trained obtaining informed consent remotely. The data manager will review all forms as well as what data is allowable to retain with research coordinators. Role play of recruitment, enrollment and informed consent process by study PI or their designee before study begins, followed by planned observation for first few enrollments and then periodically throughout the study. Additional details regarding recruitment training will be stated in the MOP.

Site study team personnel will enter data (including demographics and intervention-specific questionnaires) into an EDC system that complies with HIPAA regulations, provided by the DCOC. The EDC system includes password protection and internal quality checks, such as automatic range checks, to identify data that appear inconsistent, incomplete, or inaccurate. Study personnel will enter clinical data directly from the source documents.

10.1.10.2 STUDY RECORDS RETENTION

Throughout the course of the trial, all sites will retain the source documents on site in accordance with current site-specific medical record storage procedures.

Sites must retain all trial documents in accordance with local and/or federal regulations, whichever is most stringent. Sites will not destroy any records without the written consent of the DCOC. The DCOC will inform all site investigators when they no longer need to retain these documents.

10.1.11 PROTOCOL DEVIATIONS

Protocol deviations are any instances in which study team members and site personnel (e.g., site investigator, site coordinator, etc.) do not follow the study procedures as written in the protocol or other the explicitly stated external regulations/documents mentioned within the protocol that give direction for proper study conduct, such as the trial-specific MOP. Protocol deviations are not allowed, unless the DCOC or Operational PI gives specific written permission for the deviation. Anyone who receives written permission for a protocol deviation must keep this with other study documentation. Sites must report all known and suspected deviations to the DCOC and record all deviations in the site regulatory binder. The DCOC will conduct a review and assessment of each reported deviation depending on the results of the assessment, the DCOC will ensure that there is either a simple one-time correction made or initiate a root-cause analysis to determine if a CAPA plan is necessary to implement. CAPA plans are developed with site input for bilateral accountability and acceptance.

We will provide the specific methods for handling deviations in the trial-specific MOP and/or trial-specific SOPs.

10.1.12 PUBLICATION AND DATA SHARING POLICY

We will conduct this trial in accordance with the following publication and data sharing policies and regulations:

- **NIH Public Access Policy**, which ensures that the public has access to the published results of NIH-funded research. It requires scientists to submit final peer-reviewed journal manuscripts that arise from NIH funds to the digital archive PubMed Central upon acceptance for publication.
- **ECHO ISPCTN Publications and Presentations Policy**, which ensures accurate, responsible, and efficient communication of findings from ECHO ISPCTN clinical trials. The ECHO ISPCTN Steering Committee has approved and ratified the ECHO ISPCTN Publications and Presentations Policy, which includes representatives from all site awardees, as well as representatives from the NIH and the DCOC.

NIH Data Sharing Policy and the policy on the Dissemination of NIH-Funded Clinical Trial Information and the Clinical Trials Registration and Results Information Submission Rule. We will register this trial at ClinicalTrials.gov, and we will submit trial results to ClinicalTrials.gov. In addition, we will make every attempt to publish results in peer-reviewed journals. Other researchers may request data from this trial by contacting Song Ounpraseuth, PhD, at the DCOC.

10.1.13 CONFLICT OF INTEREST POLICY

The independence of this trial from any actual or perceived influence, such as by the pharmaceutical industry, is critical. Therefore, we will disclose and manage any actual conflict of interest of persons who have a role in the design, conduct, analysis, publication, or any aspect of this trial. Furthermore, persons who have a perceived conflict of interest will be required to have such conflicts managed in a way that is appropriate to their participation in the design and conduct of this trial. The trial leadership in conjunction with the NIH ECHO office has established policies and procedures for all trial group members to disclose all conflicts of interest and will establish a mechanism for the management of all reported dualities of interest.

10.2 TABLE AND FIGURE KEY

Section	Table/Figure Title
1.2.1	Schema (Caregiver/Child Participants)
1.2.2	Schema (Study Timeline, Study Team, Provider Participants)
1.3	Schedule of Activities
1.3	Participant Activities
3	Objectives and Endpoints
5.5.2	Screening and Enrollment Procedures

6.1.1	Caregiver and Teacher RAMP Platform Workflow
6.1.1	RAMP Platform Prototype
8.3.6	Serious Adverse Event Reporting
8.4.2	Unanticipated Problem Reporting
10.1.6	Key Roles and Study Governance
10.3	Abbreviations

10.3 ABBREVIATIONS

AAP	American Academy of Pediatrics
ADHD	Attention-Deficit/Hyperactivity Disorder
AE	Adverse Event
ANCOVA	Analysis of Covariance
CAPA	Corrective and Preventative Action
CFR	Code of Federal Regulations
CRF	Case Report Form
DCOC	Data Coordinating and Operations Center
DSMB	Data Safety Monitoring Board
DSMP	Data Safety Monitoring Plan
EDC	Electronic Data Capture
EMR	Electronic Medical Record
FDA	Food and Drug Administration
FERPA	Family Educational Rights and Privacy Act
GCDMP	Good Clinical Data Management Practices
GCP	Good Clinical Practice
HIPAA	Health Insurance Portability and Accountability Act
ICH	International Council on Harmonisation
IRB	Institutional Review Board

ISPCTN	IDeA States Pediatric Clinical Trials Network
ITT	Intention-To-Treat
MOP	Manual of Procedures
NCT	National Clinical Trial
NIH	National Institutes of Health
OHRP	Office for Human Research Protections
PHI	Protected Health Information
PI	Principal Investigator
RAMP	Remote ADHD Monitoring Program
REDCap	Research Electronic Data Capture
SAE	Serious Adverse Event
SAP	Statistical Analysis Plan
SOP	Standard Operating Procedure
UAMS	University of Arkansas Medical Sciences
UPIRTSO	Unanticipated Problem Involving Risk to Subjects or Others
US	United States

11 REFERENCES

1. Wolraich ML, Bard DE, Neas B, Doffing M, Beck L. The psychometric properties of the Vanderbilt attention-deficit hyperactivity disorder diagnostic teacher rating scale in a community population. *J Dev Behav Pediatr*. Feb 2013;34(2):83-93. doi:10.1097/DBP.0b013e31827d55c3
2. Bard DE, Wolraich ML, Neas B, Doffing M, Beck L. The psychometric properties of the Vanderbilt attention-deficit hyperactivity disorder diagnostic parent rating scale in a community population. *J Dev Behav Pediatr*. Feb 2013;34(2):72-82. doi:10.1097/DBP.0b013e31827a3a22
3. Wolraich ML, Lambert W, Doffing MA, Bickman L, Simmons T, Worley K. Psychometric properties of the Vanderbilt ADHD diagnostic parent rating scale in a referred population. *J Pediatr Psychol*. Dec 2003;28(8):559-67.
4. Wolraich ML, Feurer ID, Hannah JN, Baumgaertel A, Pinnock TY. Obtaining systematic teacher reports of disruptive behavior disorders utilizing DSM-IV. *J Abnorm Child Psychol*. Apr 1998;26(2):141-52. doi:10.1023/a:1022673906401
5. Wolraich ML, Hagan JF, Jr., Allan C, et al. Clinical Practice Guideline for the Diagnosis, Evaluation, and Treatment of Attention-Deficit/Hyperactivity Disorder in Children and Adolescents. *Pediatrics*. Oct 2019;144(4)doi:10.1542/peds.2019-2528
6. Walker AF, Hall JM, Shenkman EA, et al. Geographic access to endocrinologists for Florida's publicly insured children with diabetes. *Am J Manag Care*. Mar 2018;24(4 Spec No.):SP106-SP109.
7. Chan L, Hart LG, Goodman DC. Geographic access to health care for rural Medicare beneficiaries. *J Rural Health*. Spring 2006;22(2):140-6. doi:10.1111/j.1748-0361.2006.00022.x
8. Bosanac EM, Parkinson RC, Hall DS. Geographic access to hospital care: a 30-minute travel time standard. *Med Care*. Jul 1976;14(7):616-24. doi:10.1097/00005650-197607000-00006
9. Guille C, Maldonado L, Simpson AN, et al. A Non-Randomized Trial of In-Person Versus Text/Telephone Screening, Brief Intervention and Referral to Treatment for Pregnant and Postpartum Women. *Psychiatr Res Clin Pract*. Winter 2021;3(4):172-183. doi:10.1176/appi.prcp.20210027
10. Guille C ML, Simpson A, Newman R, King C, Cortese B, Quigley E, Dietrich N, Kerr A, Aujla R, King K, Ford D, Brady K. In-person vs. Text/Telephone Screening, Brief Intervention and Referral to Treatment for Pregnant and Postpartum Women. 2020 under review;
11. Yin H, Ibe B, Parr TL, Csukas S, Jones BL, Thompson S. Factors Associated With Children Diagnosed With Attention-Deficit/Hyperactivity Disorder and 30-Day Follow-up Care With Practitioners Among Medicaid Recipients in Georgia. *Qual Manag Health Care*. Sep 24 2021;doi:10.1097/QMH.0000000000000345
12. Davis DW, Jawad K, Feygin Y, et al. Disparities in ADHD Diagnosis and Treatment by Race/Ethnicity in Youth Receiving Kentucky Medicaid in 2017. *Ethn Dis*. Winter 2021;31(1):67-76. doi:10.18865/ed.31.1.67
13. Bettenhausen JL, Winterer CM, Colvin JD. Health and Poverty of Rural Children: An Under-Researched and Under-Resourced Vulnerable Population. *Acad Pediatr*. Nov-Dec 2021;21(8S):S126-S133. doi:10.1016/j.acap.2021.08.001
14. The Office of the National Coordinator for Health Information Technology. What electronic health record implementation issues are unique to rural settings? Updated July 8, 2019. Accessed February 27, 2022, https://www.healthit.gov/faq/what-electronic-health-record-implementation-issues-are-unique-rural-settings
15. Epstein JN, Langberg JM, Lichtenstein PK, Kolb R, Altaye M, Simon JO. Use of an Internet portal to improve community-based pediatric ADHD care: a cluster randomized trial. *Pediatrics*. Nov 2011;128(5):e1201-8. doi:10.1542/peds.2011-0872
16. Guevara JP, Power TJ, Bevans K, et al. Improving Care Management in Attention-Deficit/Hyperactivity Disorder: An RCT. *Pediatrics*. Aug 2021;148(2)doi:10.1542/peds.2020-031518

17. Dent E, Hoon E, Kitson A, et al. Translating a health service intervention into a rural setting: lessons learned. *BMC Health Serv Res*. Feb 18 2016;16:62. doi:10.1186/s12913-016-1302-0
18. Chimbindi N, Birdthistle I, Shahmanesh M, et al. Translating DREAMS into practice: Early lessons from implementation in six settings. *PLoS One*. 2018;13(12):e0208243. doi:10.1371/journal.pone.0208243
19. Mittelman MS, Bartels SJ. Translating research into practice: case study of a community-based dementia caregiver intervention. *Health Aff (Millwood)*. Apr 2014;33(4):587-95. doi:10.1377/hlthaff.2013.1334
20. Visser SN, Danielson ML, Bitsko RH, et al. Trends in the parent-report of health care provider-diagnosed and medicated attention-deficit/hyperactivity disorder: United States, 2003-2011. *J Am Acad Child Adolesc Psychiatry*. Jan 2014;53(1):34-46 e2. doi:10.1016/j.jaac.2013.09.001
21. Spencer T, Noyes E, Biederman J. Telemedicine in the Management of ADHD: Literature Review of Telemedicine in ADHD. *J Atten Disord*. Jan 2020;24(1):3-9. doi:10.1177/1087054719859081
22. Surman CBH, Fried R, Rhodewalt L, Boland H. Do Pharmaceuticals Improve Driving in Individuals with ADHD? A Review of the Literature and Evidence for Clinical Practice. *CNS Drugs*. Oct 2017;31(10):857-866. doi:10.1007/s40263-017-0465-5
23. Chamakalayil S, Strasser J, Vogel M, Brand S, Walter M, Dursteler KM. Methylphenidate for Attention-Deficit and Hyperactivity Disorder in Adult Patients With Substance Use Disorders: Good Clinical Practice. *Front Psychiatry*. 2020;11:540837. doi:10.3389/fpsyt.2020.540837
24. Epstein JN, Kelleher KJ, Baum R, et al. Specific Components of Pediatricians' Medication-Related Care Predict Attention-Deficit/Hyperactivity Disorder Symptom Improvement. *J Am Acad Child Adolesc Psychiatry*. Jun 2017;56(6):483-490 e1. doi:10.1016/j.jaac.2017.03.014
25. Jensen PS, Hinshaw SP, Swanson JM, et al. Findings from the NIMH Multimodal Treatment Study of ADHD (MTA): implications and applications for primary care providers. *J Dev Behav Pediatr*. Feb 2001;22(1):60-73. doi:10.1097/00004703-200102000-00008
26. Markowitz JT, Oberdhan D, Ciesluk A, Rams A, Wigal SB. Review of Clinical Outcome Assessments in Pediatric Attention-Deficit/Hyperactivity Disorder. *Neuropsychiatr Dis Treat*. 2020;16:1619-1643. doi:10.2147/NDT.S248685
27. Epstein JN, Kelleher KJ, Baum R, et al. Variability in ADHD care in community-based pediatrics. *Pediatrics*. Dec 2014;134(6):1136-43. doi:10.1542/peds.2014-1500
28. Syed ST, Gerber BS, Sharp LK. Traveling towards disease: transportation barriers to health care access. *J Community Health*. Oct 2013;38(5):976-93. doi:10.1007/s10900-013-9681-1
29. Wielen LM, Gilchrist EC, Nowels MA, Petterson SM, Rust G, Miller BF. Not Near Enough: Racial and Ethnic Disparities in Access to Nearby Behavioral Health Care and Primary Care. *J Health Care Poor Underserved*. Aug 2015;26(3):1032-47. doi:10.1353/hpu.2015.0083
30. Patel A, Medhekar R, Ochoa-Perez M, et al. Care Provision and Prescribing Practices of Physicians Treating Children and Adolescents With ADHD. *Psychiatr Serv*. Jul 1 2017;68(7):681-688. doi:10.1176/appi.ps.201600130
31. Foster C, Schinasi D, Kan K, Macy M, Wheeler D, Curfman A. Remote Monitoring of Patient- and Family-Generated Health Data in Pediatrics. *Pediatrics*. Feb 1 2022;149(2)doi:10.1542/peds.2021-054137
32. Nelson EC, Eftimovska E, Lind C, Hager A, Wasson JH, Lindblad S. Patient reported outcome measures in practice. *BMJ*. Feb 10 2015;350:g7818. doi:10.1136/bmj.g7818
33. Poolsup N, Suksomboon N, Kyaw AM. Systematic review and meta-analysis of the effectiveness of continuous glucose monitoring (CGM) on glucose control in diabetes. *Diabetol Metab Syndr*. 2013;5:39. doi:10.1186/1758-5996-5-39
34. Epstein JN, Kelleher KJ, Baum R, et al. Impact of a Web-Portal Intervention on Community ADHD Care and Outcomes. *Pediatrics*. Aug 2016;138(2)doi:10.1542/peds.2015-4240

35. Linder LA, Ameringer S, Erickson J, Macpherson CF, Stegenga K, Linder W. Using an iPad in research with children and adolescents. *J Spec Pediatr Nurs*. Apr 2013;18(2):158-4. doi:10.1111/jspn.12023
36. Gund A, Sjoqvist BA, Wigert H, Hentz E, Lindecrantz K, Bry K. A randomized controlled study about the use of eHealth in the home health care of premature infants. *BMC Med Inform Decis Mak*. Feb 9 2013;13:22. doi:10.1186/1472-6947-13-22
37. Silva SLO, Prado RM, Abreu-Junior CH, da Silva GP, da Silva Junior GB, da Silva JLF. (10)Boron Is Mobile in Cowpea Plants. *Front Plant Sci*. 2021;12:717219. doi:10.3389/fpls.2021.717219
38. Subcommittee on Attention-Deficit/Hyperactivity D, Steering Committee on Quality I, Management, et al. ADHD: clinical practice guideline for the diagnosis, evaluation, and treatment of attention-deficit/hyperactivity disorder in children and adolescents. *Pediatrics*. Nov 2011;128(5):1007-22. doi:10.1542/peds.2011-2654
39. Statistics NCfH. Data from: National Health Interview Survey. 2019.
40. Power TJ, Michel J, Mayne S, et al. Coordinating Systems of Care Using Health Information Technology: Development of the ADHD Care Assistant. *Adv Sch Ment Health Promot*. 2016;9(3-4):201-218. doi:10.1080/1754730X.2016.1199283
41. Whitehead AL, Julious SA, Cooper CL, Campbell MJ. Estimating the sample size for a pilot randomised trial to minimise the overall trial sample size for the external pilot and main trial for a continuous outcome variable. *Stat Methods Med Res*. Jun 2016;25(3):1057-73. doi:10.1177/0962280215588241
42. Newcombe RG. Two-sided confidence intervals for the single proportion: comparison of seven methods. *Stat Med*. Apr 30 1998;17(8):857-72. doi:10.1002/(sici)1097-0258(19980430)17:8<857::aid-sim777>3.0.co;2-e
43. Baruch Y, Holtom BC. Survey response rate levels and trends in organizational research. *Human Relations*. 2008;61(8):1139-1160. doi:10.1177/0018726708094863
