# Supplementary material for: Study protocol for a pilot study for Remote ADHD Monitoring Program (RAMP) for children in rural areas
Source: PLoS One. 2025 Dec 2;20(12):e0337802. doi: 10.1371/journal.pone.0337802 (PMC12671750; doi:10.1371/journal.pone.0337802)
Supplement: S3 File — (PDF) [file pone.0337802.s003.pdf]

NICHQ Vanderbilt Assessment Scales

Used for diagnosing ADHD

NICHQ
National Institute for
Children's Health Quality

NICHQ Vanderbilt Assessment Scale—PARENT Informant

Today's Date: ___________ Child's Name: ___________________________ Date of Birth: ___________

Parent's Name: ___________________________ Parent's Phone Number: ___________________

Directions: Each rating should be considered in the context of what is appropriate for the age of your child. When completing this form, please think about your child's behaviors in the past 6 months.

Is this evaluation based on a time when the child ☐ was on medication ☐ was not on medication ☐ not sure?

Symptoms	Never	Occasionally	Often	Very Often
1. Does not pay attention to details or makes careless mistakes with, for example, homework	0	1	2	3
2. Has difficulty keeping attention to what needs to be done	0	1	2	3
3. Does not seem to listen when spoken to directly	0	1	2	3
4. Does not follow through when given directions and fails to finish activities (not due to refusal or failure to understand)	0	1	2	3
5. Has difficulty organizing tasks and activities	0	1	2	3
6. Avoids, dislikes, or does not want to start tasks that require ongoing mental effort	0	1	2	3
7. Loses things necessary for tasks or activities (toys, assignments, pencils, or books)	0	1	2	3
8. Is easily distracted by noises or other stimuli	0	1	2	3
9. Is forgetful in daily activities	0	1	2	3
10. Fidgets with hands or feet or squirms in seat	0	1	2	3
11. Leaves seat when remaining seated is expected	0	1	2	3
12. Runs about or climbs too much when remaining seated is expected	0	1	2	3
13. Has difficulty playing or beginning quiet play activities	0	1	2	3
14. Is "on the go" or often acts as if "driven by a motor"	0	1	2	3
15. Talks too much	0	1	2	3
16. Blurts out answers before questions have been completed	0	1	2	3
17. Has difficulty waiting his or her turn	0	1	2	3
18. Interrupts or intrudes in on others' conversations and/or activities	0	1	2	3
19. Argues with adults	0	1	2	3
20. Loses temper	0	1	2	3
21. Actively defies or refuses to go along with adults' requests or rules	0	1	2	3
22. Deliberately annoys people	0	1	2	3
23. Blames others for his or her mistakes or misbehaviors	0	1	2	3
24. Is touchy or easily annoyed by others	0	1	2	3
25. Is angry or resentful	0	1	2	3
26. Is spiteful and wants to get even	0	1	2	3
27. Bullies, threatens, or intimidates others	0	1	2	3
28. Starts physical fights	0	1	2	3
29. Lies to get out of trouble or to avoid obligations (ie, "cons" others)	0	1	2	3
30. Is truant from school (skips school) without permission	0	1	2	3
31. Is physically cruel to people	0	1	2	3
32. Has stolen things that have value	0	1	2	3

The information contained in this publication should not be used as a substitute for the medical care and advice of your pediatrician. There may be variations in treatment that your pediatrician may recommend based on individual facts and circumstances.

Copyright ©2002 American Academy of Pediatrics and National Initiative for Children's Healthcare Quality

Adapted from the Vanderbilt Rating Scales developed by Mark L. Wolraich, MD.

Revised - 1102

American Academy of Pediatrics

DEDICATED TO THE HEALTH OF ALL CHILDREN™

NICHQ National Institute for Children's Health Quality

McNeil Consumer & Specialty Pharmaceuticals

NICHQ Vanderbilt Assessment Scale—PARENT Informant

Today's Date: ___________ Child's Name: ___________________________ Date of Birth: ___________

Parent's Name: ___________________________ Parent's Phone Number: ___________________

Symptoms (continued)	Never	Occasionally	Often	Very Often
33. Deliberately destroys others' property	0	1	2	3
34. Has used a weapon that can cause serious harm (bat, knife, brick, gun)	0	1	2	3
35. Is physically cruel to animals	0	1	2	3
36. Has deliberately set fires to cause damage	0	1	2	3
37. Has broken into someone else's home, business, or car	0	1	2	3
38. Has stayed out at night without permission	0	1	2	3
39. Has run away from home overnight	0	1	2	3
40. Has forced someone into sexual activity	0	1	2	3
41. Is fearful, anxious, or worried	0	1	2	3
42. Is afraid to try new things for fear of making mistakes	0	1	2	3
43. Feels worthless or inferior	0	1	2	3
44. Blames self for problems, feels guilty	0	1	2	3
45. Feels lonely, unwanted, or unloved; complains that "no one loves him or her"	0	1	2	3
46. Is sad, unhappy, or depressed	0	1	2	3
47. Is self-conscious or easily embarrassed	0	1	2	3

Performance	Excellent	Above Average	Average	Somewhat of a Problem	Problematic
48. Overall school performance	1	2	3	4	5
49. Reading	1	2	3	4	5
50. Writing	1	2	3	4	5
51. Mathematics	1	2	3	4	5
52. Relationship with parents	1	2	3	4	5
53. Relationship with siblings	1	2	3	4	5
54. Relationship with peers	1	2	3	4	5
55. Participation in organized activities (eg, teams)	1	2	3	4	5

Comments:

For Office Use Only

Total number of questions scored 2 or 3 in questions 1–9: ___________

Total number of questions scored 2 or 3 in questions 10–18: ___________

Total Symptom Score for questions 1–18: ___________

Total number of questions scored 2 or 3 in questions 19–26: ___________

Total number of questions scored 2 or 3 in questions 27–40: ___________

Total number of questions scored 2 or 3 in questions 41–47: ___________

Total number of questions scored 4 or 5 in questions 48–55: ___________

Average Performance Score: ___________

NICHQ Vanderbilt Assessment Scale—TEACHER Informant

Teacher's Name: ______________________ Class Time: ______________ Class Name/Period: ______________

Today's Date: ________ Child's Name: ______________________ Grade Level: ______________________

Directions: Each rating should be considered in the context of what is appropriate for the age of the child you are rating and should reflect that child's behavior since the beginning of the school year. Please indicate the number of weeks or months you have been able to evaluate the behaviors: ________.

Is this evaluation based on a time when the child ☐ was on medication ☐ was not on medication ☐ not sure?

Symptoms	Never	Occasionally	Often	Very Often
1. Fails to give attention to details or makes careless mistakes in schoolwork	0	1	2	3
2. Has difficulty sustaining attention to tasks or activities	0	1	2	3
3. Does not seem to listen when spoken to directly	0	1	2	3
4. Does not follow through on instructions and fails to finish schoolwork (not due to oppositional behavior or failure to understand)	0	1	2	3
5. Has difficulty organizing tasks and activities	0	1	2	3
6. Avoids, dislikes, or is reluctant to engage in tasks that require sustained mental effort	0	1	2	3
7. Loses things necessary for tasks or activities (school assignments, pencils, or books)	0	1	2	3
8. Is easily distracted by extraneous stimuli	0	1	2	3
9. Is forgetful in daily activities	0	1	2	3
10. Fidgets with hands or feet or squirms in seat	0	1	2	3
11. Leaves seat in classroom or in other situations in which remaining seated is expected	0	1	2	3
12. Runs about or climbs excessively in situations in which remaining seated is expected	0	1	2	3
13. Has difficulty playing or engaging in leisure activities quietly	0	1	2	3
14. Is "on the go" or often acts as if "driven by a motor"	0	1	2	3
15. Talks excessively	0	1	2	3
16. Blurts out answers before questions have been completed	0	1	2	3
17. Has difficulty waiting in line	0	1	2	3
18. Interrupts or intrudes on others (eg, butts into conversations/games)	0	1	2	3
19. Loses temper	0	1	2	3
20. Actively defies or refuses to comply with adult's requests or rules	0	1	2	3
21. Is angry or resentful	0	1	2	3
22. Is spiteful and vindictive	0	1	2	3
23. Bullies, threatens, or intimidates others	0	1	2	3
24. Initiates physical fights	0	1	2	3
25. Lies to obtain goods for favors or to avoid obligations (eg, "cons" others)	0	1	2	3
26. Is physically cruel to people	0	1	2	3
27. Has stolen items of nontrivial value	0	1	2	3
28. Deliberately destroys others' property	0	1	2	3
29. Is fearful, anxious, or worried	0	1	2	3
30. Is self-conscious or easily embarrassed	0	1	2	3
31. Is afraid to try new things for fear of making mistakes	0	1	2	3

The recommendations in this publication do not indicate an exclusive course of treatment or serve as a standard of medical care. Variations, taking into account individual circumstances, may be appropriate.

Copyright ©2002 American Academy of Pediatrics and National Initiative for Children's Healthcare Quality

Adapted from the Vanderbilt Rating Scales developed by Mark L. Wolraich, MD.

Revised - 0303

American Academy of Pediatrics

DEDICATED TO THE HEALTH OF ALL CHILDREN™

NICHQ National Institute for Children's Health Quality

McNeil Consumer & Specialty Pharmaceuticals

Teacher's Name: ______________________________ Class Time: __________________ Class Name/Period: ______________

Today's Date: __________ Child's Name: ______________________________ Grade Level: ______________________________

Symptoms (continued)	Never	Occasionally	Often	Very Often
32. Feels worthless or inferior	0	1	2	3
33. Blames self for problems; feels guilty	0	1	2	3
34. Feels lonely, unwanted, or unloved; complains that "no one loves him or her"	0	1	2	3
35. Is sad, unhappy, or depressed	0	1	2	3

**Performance** *Academic Performance*	Excellent	Above Average	Average	Somewhat of a Problem	Problematic
36. Reading	1	2	3	4	5
37. Mathematics	1	2	3	4	5
38. Written expression	1	2	3	4	5

Classroom Behavioral Performance	Excellent	Above Average	Average	Somewhat of a Problem	Problematic
39. Relationship with peers	1	2	3	4	5
40. Following directions	1	2	3	4	5
41. Disrupting class	1	2	3	4	5
42. Assignment completion	1	2	3	4	5
43. Organizational skills	1	2	3	4	5

Comments:

Please return this form to: ____

Mailing address: ____

____

Fax number: ____

For Office Use Only

Total number of questions scored 2 or 3 in questions 1–9: ______________

Total number of questions scored 2 or 3 in questions 10–18: ______________

Total Symptom Score for questions 1–18: ______________

Total number of questions scored 2 or 3 in questions 19–28: ______________

Total number of questions scored 2 or 3 in questions 29–35: ______________

Total number of questions scored 4 or 5 in questions 36–43: ______________

Average Performance Score: ______________

D5 NICHQ Vanderbilt Assessment Follow-up—PARENT Informant

Today's Date: ___________ Child's Name: ____ Date of Birth: ______________

Parent's Name: ____ Parent's Phone Number: ____________________________

Directions: Each rating should be considered in the context of what is appropriate for the age of your child. Please think about your child's behaviors since the last assessment scale was filled out when rating his/her behaviors.

Is this evaluation based on a time when the child ☐ was on medication ☐ was not on medication ☐ not sure?

Symptoms	Never	Occasionally	Often	Very Often
1. Does not pay attention to details or makes careless mistakes with, for example, homework	0	1	2	3
2. Has difficulty keeping attention to what needs to be done	0	1	2	3
3. Does not seem to listen when spoken to directly	0	1	2	3
4. Does not follow through when given directions and fails to finish activities (not due to refusal or failure to understand)	0	1	2	3
5. Has difficulty organizing tasks and activities	0	1	2	3
6. Avoids, dislikes, or does not want to start tasks that require ongoing mental effort	0	1	2	3
7. Loses things necessary for tasks or activities (toys, assignments, pencils, or books)	0	1	2	3
8. Is easily distracted by noises or other stimuli	0	1	2	3
9. Is forgetful in daily activities	0	1	2	3
10. Fidgets with hands or feet or squirms in seat	0	1	2	3
11. Leaves seat when remaining seated is expected	0	1	2	3
12. Runs about or climbs too much when remaining seated is expected	0	1	2	3
13. Has difficulty playing or beginning quiet play activities	0	1	2	3
14. Is "on the go" or often acts as if "driven by a motor"	0	1	2	3
15. Talks too much	0	1	2	3
16. Blurts out answers before questions have been completed	0	1	2	3
17. Has difficulty waiting his or her turn	0	1	2	3
18. Interrupts or intrudes in on others' conversations and/or activities	0	1	2	3

Performance	Excellent	Above Average	Average	Somewhat of a Problem	Problematic
19. Overall school performance	1	2	3	4	5
20. Reading	1	2	3	4	5
21. Writing	1	2	3	4	5
22. Mathematics	1	2	3	4	5
23. Relationship with parents	1	2	3	4	5
24. Relationship with siblings	1	2	3	4	5
25. Relationship with peers	1	2	3	4	5
26. Participation in organized activities (eg, teams)	1	2	3	4	5

The information contained in this publication should not be used as a substitute for the medical care and advice of your pediatrician. There may be variations in treatment that your pediatrician may recommend based on individual facts and circumstances.

Copyright ©2002 American Academy of Pediatrics and National Initiative for Children's Healthcare Quality

Adapted from the Vanderbilt Rating Scales developed by Mark L. Wolraich, MD.

Revised - 0303

American Academy of Pediatrics

DEDICATED TO THE HEALTH OF ALL CHILDREN™

NICHQ National Institute for Children's Health Quality

McNeil Consumer & Specialty Pharmaceuticals

HE0352

Today's Date: ___________ Child's Name: ____ Date of Birth: ______________

Parent's Name: ____ Parent's Phone Number: ____________________________

**Side Effects:** Has your child experienced any of the following side effects or problems in the past week?	**Are these side effects currently a problem?**			
	**None**	**Mild**	**Moderate**	**Severe**
Headache				
Stomachache				
Change of appetite—explain below				
Trouble sleeping				
Irritability in the late morning, late afternoon, or evening—explain below				
Socially withdrawn—decreased interaction with others				
Extreme sadness or unusual crying				
Dull, tired, listless behavior				
Tremors/feeling shaky				
Repetitive movements, tics, jerking, twitching, eye blinking—explain below				
Picking at skin or fingers, nail biting, lip or cheek chewing—explain below				
Sees or hears things that aren't there				

Explain/Comments:

For Office Use Only

Total Symptom Score for questions 1–18: ______________________________

Average Performance Score for questions 19–26: ______________________________

Adapted from the Pittsburgh side effects scale, developed by William E. Pelham, Jr, PhD.

American Academy of Pediatrics

DEDICATED TO THE HEALTH OF ALL CHILDREN™

NICHQ National Institute for Children's Health Quality

McNeil Consumer & Specialty Pharmaceuticals

D6 NICHQ Vanderbilt Assessment Follow-up—TEACHER Informant

Teacher's Name: ______________________ Class Time: ______________ Class Name/Period: ____________

Today's Date: ________ Child's Name: ______________________ Grade Level: ______________________

Directions: **Each rating should be considered in the context of what is appropriate for the age of the child you are rating and should reflect that child's behavior since the last assessment scale was filled out. Please indicate the number of weeks or months you have been able to evaluate the behaviors: ________.**

Is this evaluation based on a time when the child ☐ **was on medication** ☐ **was not on medication** ☐ **not sure?**

Symptoms	Never	Occasionally	Often	Very Often
1. Does not pay attention to details or makes careless mistakes with, for example, homework	0	1	2	3
2. Has difficulty keeping attention to what needs to be done	0	1	2	3
3. Does not seem to listen when spoken to directly	0	1	2	3
4. Does not follow through when given directions and fails to finish activities (not due to refusal or failure to understand)	0	1	2	3
5. Has difficulty organizing tasks and activities	0	1	2	3
6. Avoids, dislikes, or does not want to start tasks that require ongoing mental effort	0	1	2	3
7. Loses things necessary for tasks or activities (toys, assignments, pencils, or books)	0	1	2	3
8. Is easily distracted by noises or other stimuli	0	1	2	3
9. Is forgetful in daily activities	0	1	2	3
10. Fidgets with hands or feet or squirms in seat	0	1	2	3
11. Leaves seat when remaining seated is expected	0	1	2	3
12. Runs about or climbs too much when remaining seated is expected	0	1	2	3
13. Has difficulty playing or beginning quiet play activities	0	1	2	3
14. Is "on the go" or often acts as if "driven by a motor"	0	1	2	3
15. Talks too much	0	1	2	3
16. Blurts out answers before questions have been completed	0	1	2	3
17. Has difficulty waiting his or her turn	0	1	2	3
18. Interrupts or intrudes in on others' conversations and/or activities	0	1	2	3

Performance	Excellent	Above Average	Average	Somewhat of a Problem	Problematic
19. Reading	1	2	3	4	5
20. Mathematics	1	2	3	4	5
21. Written expression	1	2	3	4	5
22. Relationship with peers	1	2	3	4	5
23. Following direction	1	2	3	4	5
24. Disrupting class	1	2	3	4	5
25. Assignment completion	1	2	3	4	5
26. Organizational skills	1	2	3	4	5

The recommendations in this publication do not indicate an exclusive course of treatment or serve as a standard of medical care. Variations, taking into account individual circumstances, may be appropriate.

Copyright ©2002 American Academy of Pediatrics and National Initiative for Children's Healthcare Quality

Adapted from the Vanderbilt Rating Scales developed by Mark L. Wolraich, MD.

Revised - 0303

American Academy of Pediatrics

DEDICATED TO THE HEALTH OF ALL CHILDREN™

NICHQ National Institute for Children's Health Quality

McNeil Consumer & Specialty Pharmaceuticals

HE0353

Teacher's Name: ______________________________ Class Time: ___________________ Class Name/Period: ________________

Today's Date: ___________ Child's Name: ______________________________ Grade Level: ______________________________

**Side Effects:** Has the child experienced any of the following side effects or problems in the past week?	**Are these side effects currently a problem?**			
	**None**	**Mild**	**Moderate**	**Severe**
Headache				
Stomachache				
Change of appetite—explain below				
Trouble sleeping				
Irritability in the late morning, late afternoon, or evening—explain below				
Socially withdrawn—decreased interaction with others				
Extreme sadness or unusual crying				
Dull, tired, listless behavior				
Tremors/feeling shaky				
Repetitive movements, tics, jerking, twitching, eye blinking—explain below				
Picking at skin or fingers, nail biting, lip or cheek chewing—explain below				
Sees or hears things that aren't there				

Explain/Comments:

For Office Use Only

Total Symptom Score for questions 1–18: ______________________________

Average Performance Score: ______________________________

Please return this form to: ______________________________

Mailing address: ______________________________

____

Fax number: ______________________________

Adapted from the Pittsburgh side effects scale, developed by William E. Pelham, Jr, PhD.

Scoring Instructions for the NICHQ Vanderbilt Assessment Scales

These scales should NOT be used alone to make any diagnosis. You must take into consideration information from multiple sources. Scores of 2 or 3 on a single Symptom question reflect *often-occurring* behaviors. Scores of 4 or 5 on Performance questions reflect problems in performance.

The initial assessment scales, parent and teacher, have 2 components: symptom assessment and impairment in performance. On both the parent and teacher initial scales, the symptom assessment screens for symptoms that meet criteria for both inattentive (items 1–9) and hyperactive ADHD (items 10–18).

To meet *DSM-IV* criteria for the diagnosis, one must have at least 6 positive responses to either the inattentive 9 or hyperactive 9 core symptoms, or both. A positive response is a 2 or 3 (often, very often) (you could draw a line straight down the page and count the positive answers in each subsegment). There is a place to record the number of positives in each subsegment, and a place for total score for the first 18 symptoms (just add them up).

The initial scales also have symptom screens for 3 other co-morbidities—oppositional-defiant, conduct, and anxiety/depression. These are screened by the number of positive responses in each of the segments separated by the "squares." The specific item sets and numbers of positives required for each co-morbid symptom screen set are detailed below.

The second section of the scale has a set of performance measures, scored 1 to 5, with 4 and 5 being somewhat of a problem/problematic. To meet criteria for ADHD there must be at least one item of the Performance set in which the child scores a 4 or 5; ie, there must be impairment, not just symptoms to meet diagnostic criteria. The sheet has a place to record the number of positives (4s, 5s) and an Average Performance Score—add them up and divide by number of Performance criteria answered.

Parent Assessment Scale

Predominantly Inattentive subtype
- Must score a 2 or 3 on 6 out of 9 items on questions 1–9 AND
- Score a 4 or 5 on any of the Performance questions 48–55

Predominantly Hyperactive/Impulsive subtype
- Must score a 2 or 3 on 6 out of 9 items on questions 10–18 AND
- Score a 4 or 5 on any of the Performance questions 48–55

ADHD Combined Inattention/Hyperactivity
- Requires the above criteria on both inattention and hyperactivity/impulsivity

Oppositional-Defiant Disorder Screen
- Must score a 2 or 3 on 4 out of 8 behaviors on questions 19–26 AND
- Score a 4 or 5 on any of the Performance questions 48–55

Conduct Disorder Screen
- Must score a 2 or 3 on 3 out of 14 behaviors on questions 27–40 AND
- Score a 4 or 5 on any of the Performance questions 48–55

Anxiety/Depression Screen
- Must score a 2 or 3 on 3 out of 7 behaviors on questions 41–47 AND
- Score a 4 or 5 on any of the Performance questions 48–55

Teacher Assessment Scale

Predominantly Inattentive subtype
- Must score a 2 or 3 on 6 out of 9 items on questions 1–9 AND
- Score a 4 or 5 on any of the Performance questions 36–43

Predominantly Hyperactive/Impulsive subtype
- Must score a 2 or 3 on 6 out of 9 items on questions 10–18 AND
- Score a 4 or 5 on any of the Performance questions 36–43

ADHD Combined Inattention/Hyperactivity
- Requires the above criteria on both inattention and hyperactivity/impulsivity

Oppositional-Defiant/Conduct Disorder Screen
- Must score a 2 or 3 on 3 out of 10 items on questions 19–28 AND
- Score a 4 or 5 on any of the Performance questions 36–43

Anxiety/Depression Screen
- Must score a 2 or 3 on 3 out of 7 items on questions 29–35 AND
- Score a 4 or 5 on any of the Performance questions 36–43

The parent and teacher follow-up scales have the first 18 core ADHD symptoms, not the co-morbid symptoms. The section segment has the same Performance items and impairment assessment as the initial scales, and then has a side-effect reporting scale that can be used to both assess and monitor the presence of adverse reactions to medications prescribed, if any.

Scoring the follow-up scales involves only calculating a total symptom score for items 1–18 that can be tracked over time, and the average of the Performance items answered as measures of improvement over time with treatment.

Parent Assessment Follow-up

- Calculate Total Symptom Score for questions 1–18.
- Calculate Average Performance Score for questions 19–26.

Teacher Assessment Follow-up

- Calculate Total Symptom Score for questions 1–18.
- Calculate Average Performance Score for questions 19–26.

The recommendations in this publication do not indicate an exclusive course of treatment or serve as a standard of medical care. Variations, taking into account individual circumstances, may be appropriate.

Copyright ©2002 American Academy of Pediatrics and National Initiative for Children's Healthcare Quality

American Academy of Pediatrics

DEDICATED TO THE HEALTH OF ALL CHILDREN™

NICHQ National Institute for Children's Health Quality

McNeil
